# Therapeutic potential of natural compounds from medicinal and food homology substances targeting gut microbiota in lipid metabolism disorders

**DOI:** 10.3389/fmicb.2026.1809114

**Published:** 2026-05-15

**Authors:** Qinjing Xia, Jixuan Xin, Yue Mu, Mingquan Li, Juan Yao, Xijingyi Xiao, Xiuge Wang, Xiaochao Gang

**Affiliations:** 1 Changchun University of Chinese Medicine, Changchun, Jilin, China; 2 The Third Affiliated Hospital of Changchun University of Chinese Medicine, Changchun, Jilin, China; 3 Departments of Acupuncture and Massage, Changchun University of Chinese Medicine, Changchun, Jilin, China; 4 The Affiliated Hospital of Changchun University of Chinese Medicine, Changchun, Jilin, China

**Keywords:** gut microbiota, lipid metabolism, medicinal and food homology, polyphenols, polysaccharides, saponins, terpenoids, alkaloids

## Abstract

Dyslipidemia contributes to chronic diseases such as non-alcoholic fatty liver disease (NAFLD), type 2 diabetes (T2DM), and obesity. Emerging evidence highlights gut dysbiosis as a key driver of abnormal lipid metabolism. This review examines how natural bioactive compounds from medicinal and food homology (MFH) substances regulate lipid metabolism by modulating the gut microbiome. It summarizes evidence on the modification of the microbiota-lipid metabolism axis by natural compounds from MFH substances and discusses the limitations of applications and their promise for preventing and treating metabolic diseases. By capitalizing on these microbiota-mediated effects, natural compounds may serve as a beneficial natural resource for adjusting lipid metabolism.

## 1 Introduction

Lipids, encompassing triglycerides (TG), phospholipids, sterols, and other lipoids, comprise 10–20% of the human body. Lipids are important energy sources and provide essential fatty acids (FAs). Furthermore, they also serve as fundamental structural elements of cells and tissues and play crucial roles in signaling and regulation.

Lipid metabolism maintains energy balance, membrane structure, and signal transduction (Ping et al., 2025). FAs, the basic lipid units, are key components of phospholipids and sphingolipids, influencing membrane properties and cellular regulation. During energy production, FAs are converted into acetyl-CoA via β-oxidation in mitochondria and peroxisomes. Acetyl-CoA then enters the tricarboxylic acid (TCA) cycle, driving ATP synthesis through oxidative phosphorylation (Dyall et al., 2022; Huang et al., 2022; Wedan et al., 2024).

Dyslipidemia is the pathological accumulation or imbalance of lipids and their metabolites in circulation and tissues. It is a direct cause of the development and advancement of several disorders, such as metabolic syndrome (MetS), obesity, T2DM, NAFLD, and hyperlipidemia (Petrenko et al., 2023). Consequently, lipid metabolic homeostasis is highly important in avoiding chronic diseases and enhancing overall health. It is noteworthy that malfunctions in key enzymes, related proteins, epigenetic regulation, and specific amino acids are vital factors that disrupt lipid metabolism and impair its normal functioning (Wu Y. L. et al., 2023; Shan et al., 2025; Zhou et al., 2025).

Lipid metabolic disorders are intimately linked with the gut microbiota’s regulating function, which influences human health and illness. The millions of trillions of gut bacteria within the human body dynamically respond to physiological and pathological alterations and have extensive, multifaceted effects on the metabolism of lipids. The gut microbiota serves as the body’s second liver. It harbors a vast array of enzyme systems that allow it to break down and transform dietary lipid components. Conversely, the microbiota can *de novo* biosynthesize and biotransform lipids into diverse lipid molecules with key structural and signaling roles, including sphingolipids, glycolipids, and phospholipids. These microbial products regulate host lipid uptake, storage, and utilization through metabolic and immunological pathways (Cai et al., 2022; Brown et al., 2023). Consequently, gut dysbiosis can disrupt the balance between metabolic and immunological stability, ultimately leading to the development of metabolic diseases (Jardon et al., 2022).

MFH substances possess nutritional and therapeutic properties, which is why they are a significant component of traditional Chinese medicine. This was first documented in the Huangdi Neijing, the earliest medical treatise in China. MFH substances contain numerous natural bioactive substances, which are polysaccharides, polyphenols, saponins, and alkaloids, as well as essential oils, volatile oils, and terpenoids. These ingredients provide MFH substances with multifaceted efficacy, including antibacterial, antioxidant, anti-inflammatory, anticancer, and antiviral impacts, at relatively high levels of safety (Lu et al., 2022; Cui et al., 2024). Over the past few years, the intestinal microbiota has come to prominence as a new research target for MFH substances, largely because of their capacity to remodel gut microbial composition and function (Zhang C. et al., 2024). Evidence indicates that natural compounds from MFH substances effectively stimulate the growth of healthy gut microbes, regulate gut dysbiosis, and modulate lipid metabolism, making them a valuable intervention for maintaining human health (Xia et al., 2025).

This review evaluates the therapeutic potential of natural compounds from MFH substances for treating lipid-metabolism diseases by regulating the gut microbiota. It describes the interaction mechanisms between relevant natural compounds, the gut microbiota’s structure, their products, and lipid metabolism, thereby offering a theoretical basis for the creation of novel medications, functional food, and additional treatments.

## 2 Search strategy and inclusion criteria

### 2.1 Search strategy

This review employed a narrative review approach, conducting a literature search through online databases (PubMed, ScienceDirect, and other library resources). Search terms were combined using “AND”. Keywords were grouped into three categories: (1) terms related to the concept of “medicinal and food homology” (e.g., “food as medicine,” “food-medicine substances,” and a list of specific substances such as Angelica sinensis, Pueraria lobata, Coix lacryma-jobi, ginger, goji berries, poria, reishi mushroom, platycodon, Codonopsis, etc.); (2) terms related to the gut microbiome (“gut microbiome” or “gut flora”); (3) terms related to lipid metabolism (“lipid metabolism,” “dyslipidemia,” “obesity,” “NAFLD,” or “T2DM”).

### 2.2 Inclusion criteria

**FIGURE 1:**
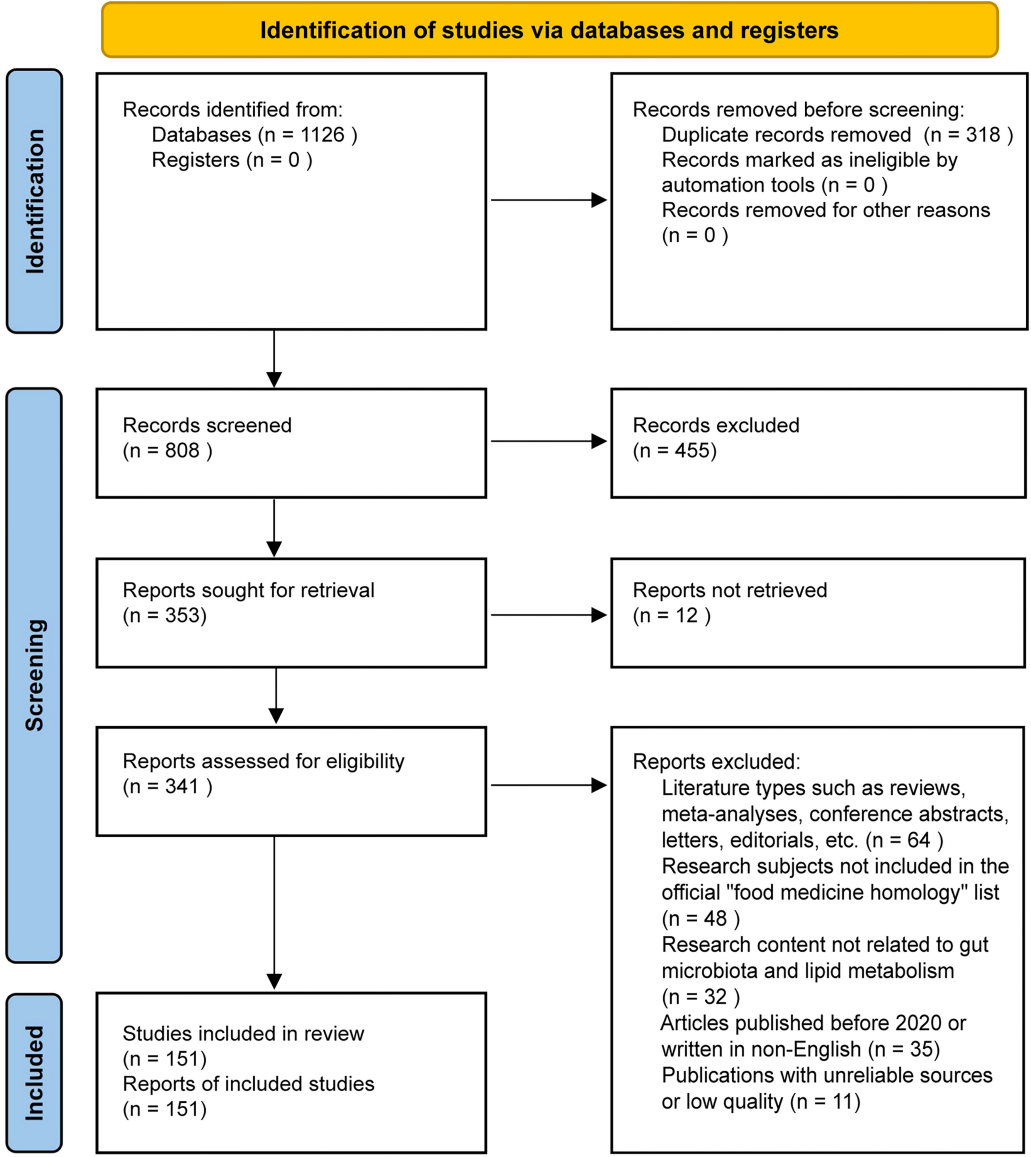
Literature selection process diagram (PRISMA style).

This article provides a PRISMA-style flowchart (Figure 1) to enhance the transparency of the literature selection process. This figure serves solely to illustrate the literature search and screening process for this narrative review and does not imply that this review followed a formal systematic review methodology. Inclusion criteria included: (1) original *in vivo* or *in vitro* studies (2020–2025, English); (2) use of natural compounds from China’s officially listed “food-medicine” substances; (3) focus on gut microbiota–lipid metabolism interactions; (4) published in peer-reviewed, high-quality journals. Exclusion criteria included: (1) reviews, meta-analyses, conference abstracts, letters, editorials, or similar document types; (2) research subjects were not on the official “food-medicine” list; (3) studies that do not examine the relationship between gut microbiota and lipid metabolism; (4) articles published before 2020 or not written in English; (5) publications from unreliable or low-quality sources.

## 3 Gut microbiota: the bridge to host lipid metabolic health

### 3.1 Lipid metabolic abnormalities in metabolic diseases

Obesity is a persistent metabolic disorder characterized by excessive fat accumulation. It is caused by continued energy intake exceeding energy use, leading to adipocyte hyperplasia and hypertrophy, increased inflammatory responses, impaired extracellular matrix remodeling and fibrosis, and abnormal adipokine release (Unamuno et al., 2018). In healthy environments, fat stores support metabolism by promoting energy burning via thermogenesis, glucose and FAs uptake, and the release of a wide variety of bioactive molecules (hormones, metabolites, and genetic material). Adipose tissue dysfunction promotes the occurrence and development of metabolic disorders by inducing inflammation, elevating free fatty acid levels, promoting ectopic lipid formation, and altering the adipose tissue secretory profile (Wang et al., 2022b; An et al., 2023; Xu et al., 2024). Existing therapeutic strategies for obesity include medications such as incretin mimetics, increased energy expenditure by stimulating brown and beige adipocytes pharmacologically, and modulation of specific adipokines.

T2DM, a metabolic disease closely related to obesity, is characterized by insulin resistance (IR) and relative insulin deficiency. It is clinically linked to chronic hyperglycemia, hyperinsulinemia, dyslipidemia, chronic low-level inflammation, and elevated risk of obesity (Westman, 2021; Luciani et al., 2024). T2DM is recognized as a syndrome with several sub-phenotypes, including mitochondrial dysfunction affecting cellular bioenergetics, adipose tissue dysfunction disrupting lipid storage and adipokine secretion, and cytokine-mediated inflammatory responses promoting systemic inflammation. Among the most common phenotypes is dyslipidemia, characterized by higher plasma TG and low-density lipoprotein cholesterol (LDL-C) and decreased high-density lipoprotein cholesterol (HDL-C) (Kane et al., 2021; Ağagündüz et al., 2023). Its pathophysiology involves mitochondrial dysfunction, endoplasmic reticulum stress, altered fatty acid metabolism, and impaired β-oxidation (Lu et al., 2024). Although T2DM is incurable, lifestyle interventions and pharmacologic antihyperglycemic agents can attenuate disease progression and reduce complication risks.

**FIGURE 2:**
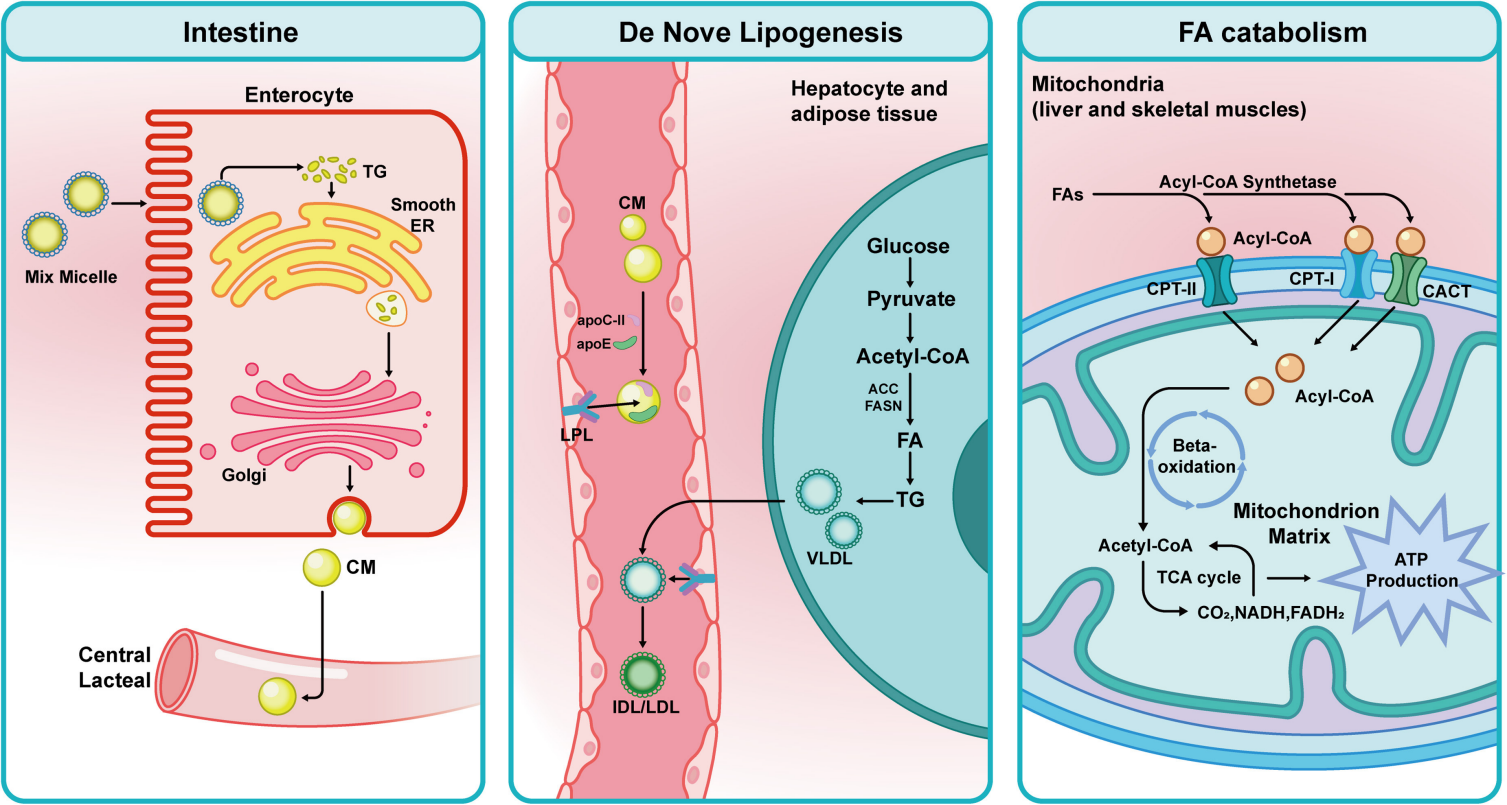
The gut microbiota participates in lipid metabolism. Digestive enzymes hydrolyze dietary fats to create mixed micelles that enter intestinal epithelial cells. These are re-esterified into triglycerides, phospholipids, and other compounds. They pass through the central lacteals after being further processed by the Golgi apparatus into chylomicrons (CM). CM attaches to apolipoproteins (apoC-II and apoE) after entering the bloodstream through lymphatic fluid. Lipoprotein lipase (LPL) converts its core triglycerides into FAs and glycerol. The crucial sites of FAs production are liver and adipose tissue. Glucose undergoes glycolysis to produce pyruvate, which enters the cytoplasm and is transformed into acetyl-CoA. Acetyl-CoA is transformed into FAs under the regulation of FASN and ACC. VLDL, which are released into the bloodstream, are created when FAs combine with apolipoproteins after being esterified into TG. LPL hydrolyzes VLDL, progressively changing it into intermediate-density lipoproteins/low-density lipoproteins (IDL/LDL). The mitochondria of the liver and skeletal muscle are primarily where fatty acid breakdown occurs. Carnitine palmitoyltransferase I (CPT-I), carnitine acyl-L-carnitine translocase (CACT), and carnitine palmitoyltransferase II (CPT-II) transport fatty acids into mitochondria after they are first converted to acyl-CoA. Malonyl-CoA, glucose, and PPARα control the expression of these three important enzymes. Through β-oxidation, acetyl-CoA is produced, entering the citric acid cycle and oxidative phosphorylation to generate carbon dioxide, water, and ATP for energy. Lipid metabolism disorders can result from abnormalities in these pathways.

NAFLD is a systemic disease caused by obesity, T2DM, and metabolic dysfunction. It is marked by intestinal dysbiosis, IR, lipotoxicity, hepatic lipid buildup, and persistent inflammation. The pathological basis of this situation is hepatic steatosis, which results from an imbalance between fatty acid intake from the plasma and *de novo* lipogenesis (DNL) in the liver, which cannot be offset by fatty acid oxidation, TG, and very low-density lipoprotein (VLDL) production (Pafili and Roden, 2021; Esler and Cohen, 2024). After some time, hepatocellular carcinoma, cirrhosis, liver fibrosis, and non-alcoholic steatohepatitis (NASH) can develop from NAFLD (Tilg et al., 2021). Along with improved knowledge of the causes of NAFLD, forms of therapy have increased to include lifestyle modification, bariatric surgery, and an assortment of pharmaceutical treatments, including hypoglycemic drugs and anti-inflammatory drugs (Figure 2).

### 3.2 The gut microbiota’s dynamic characteristics and its critical role in metabolic health

The symbiotic relationship between the gut microbiota and host metabolic health hinges on two essential ecological properties: stability and flexibility. While certain genera like *Bifidobacterium*, *Enterobacter*, *Faecalibacterium*, and *Bacteroides* remain relatively stable, the overall gut composition is highly plastic, responding dynamically to genetic, nutritional, behavioral, and environmental factors. As a key factor in gut ecology, diet plays a decisive role in predetermining the structure and function, which profoundly impact host metabolism and health (Zmora et al., 2019; Klimenko et al., 2022; Ross et al., 2024; Schoultz et al., 2025). Included among them is the observation that the gut microbiome is more predictive of postprandial lipid and insulin levels than of glucose levels. Take *Prevotella copri*, for instance: The presence of this bacterium might help maintain glucose homeostasis and host metabolic conditions. Associations between diet and microbiota, and between blood biomarkers and circulating lipid levels, are stronger than those with glycemic indices. It demonstrates that the gut microbiome may be significantly more critical to circulating lipid concentrations than to glucose metabolism (Asnicar et al., 2021).

A healthy gut microbiota, dominated by obligate anaerobes, metabolizes dietary carbohydrates into a variety of metabolites, including bile acids (BAs), short-chain fatty acids (SCFAs), imidazole propionate, lipopolysaccharides (LPS), flagellins, and branched-chain amino acids (BCAAs) (Canfora et al., 2019; Baars et al., 2024). SCFAs and BAs improve metabolic health—including insulin sensitivity and lipid homeostasis—by maintaining intestinal stability and regulating adipose and hepatic functions. This effect slows the progression of lipid-metabolic diseases.

Patients with lipid metabolic disorders frequently have lower gut microbial diversity, fewer obligate anaerobes, and a higher proportion of facultative anaerobes (Canfora et al., 2019). The altered taxa in NAFLD patients include *Ruminococcus*, *Eubacterium hallii*, *Eggerthella lenta*, *Clostridium bolteae*, *Intestinibacter bartlettii*, and *Roseburia intestinalis* (Nychas et al., 2025). Similarly, several studies report lower numbers of bacteria that produce butyrate, like *Faecalibacterium*, *Clostridium*, and *Akkermansia*, in T2DM patients, alongside an increased number of *Escherichia coli* (Baars et al., 2024). *Proteobacteria*, *Lactobacillus*, and *Fusobacterium* are usually more abundant in obese people, whereas *Faecalibacterium*, *Akkermansia*, and *Alistipes* are greatly reduced (Xuan et al., 2023; Li L. et al., 2024; Saad and Santos, 2025).

### 3.3 Bidirectional interactions between MFH substances and the gut microbiome and their health effects

Based on their biological origin, MFH substances comprise three types: fungi, animals, and plants. Plant-derived accounts for more than 90% and plays the most important role in practical applications. Consuming MFH substances can help manage the makeup of the human gut flora. For example, randomized crossover trials demonstrated targeted remodeling of the gut microbiome following consumption of inulin from chicory root (Watson et al., 2019; Reimer et al., 2020; Kiewiet et al., 2021; Horasan Sagbasan et al., 2024), almonds (Creedon A. C. et al., 2022; Creedon A. et al., 2022), cassia seeds (Narrowe et al., 2024), coix seeds (Jinnouchi et al., 2021), ginger (Wang X. et al., 2020; Crichton et al., 2023), turmeric (Peron et al., 2020), and American ginseng (Bell et al., 2022). Beneficial bacteria, including *Phascolarctobacterium*, *Bifidobacterium*, *Faecalibacterium*, *Lactobacillus*, *Akkermansia*, and *Alistipes*, were found to be more abundant in subjects who consumed MFH, whereas certain microbiota associated with dysbiosis, such as *Escherichia* and pro-inflammatory *Ruminococcus_1* and *Ruminococcus_2*, were significantly reduced in abundance.

**FIGURE 3:**
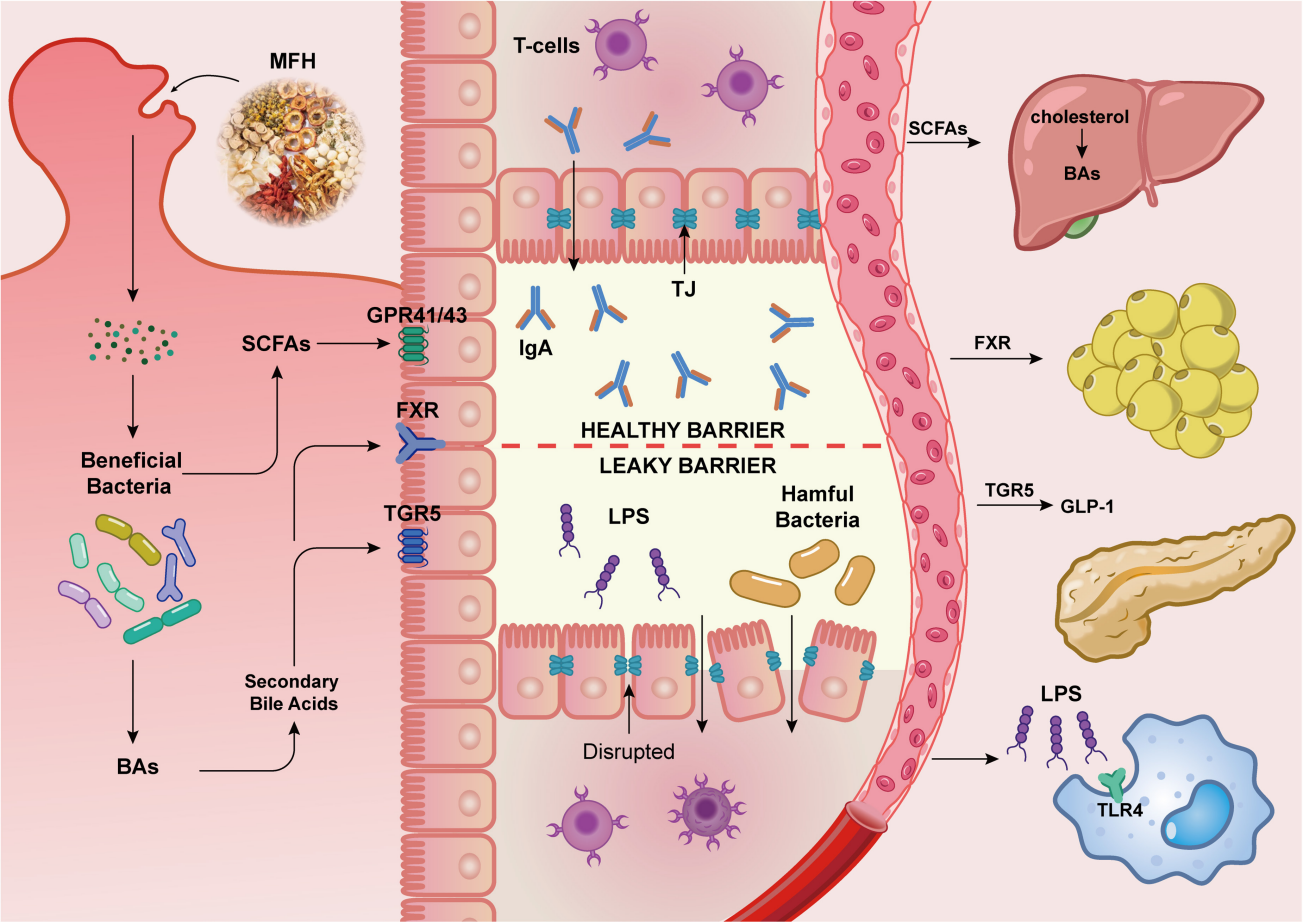
The function of microbiota, metabolites, and the intestinal barrier in maintaining metabolic homeostasis. The intestinal barrier efficiently prevents toxins, pathogenic microbes, and other macromolecules from entering the bloodstream while selectively absorbing water and nutrients. It regulates microbial balance and maintains immunological homeostasis. Hepatocytes use enzymes like cholesterol 7α-hydroxylase (CYP7A1) to convert cholesterol into primary bile acids. The microbiota in the gut converts primary bile acids into secondary bile acids. By activating nuclear receptors (like the FXR) and membrane receptors (like the GPR), bile acids function as essential metabolic signaling molecules that regulate their own synthesis, glucose homeostasis, lipid metabolism, and energy expenditure. The gut microbiota produces SCFAs through the fermentation process. Butyrate is the main energy source for colonic epithelial cells, which enhances intestinal barrier function. As acetate and propionate enter the bloodstream, they promote GLP-1 and peptide YY (PYY) release to improve satiety, as well as contribute to the regulation of cholesterol and FAs production in the liver. While the intestinal barrier is compromised, Gram-negative bacteria and their LPS enter the bloodstream through the gut wall, activating immune cells via TLR4 and causing inflammation. When abnormalities of lipid metabolism occur, natural compounds from MFH substances can help maintain lipid homeostasis by targeting gut microbiota remodeling and regulating metabolites.

In contrast, gut microbes are essential to controlling the digestion, bioavailability, and absorption of MFH components. Owing to their high dietary fiber content, MFH substances resist degradation by host enzymes within the upper gastrointestinal tract, enabling them to reach the cecum and colon intact. The gut microbiota harbors a diverse array of glycoside hydrolases (over 260 types), which convert dietary carbohydrates into numerous small-molecule bioactive compounds, thereby driving microbial fermentation. The increase in beneficial bacteria stimulates the synthesis of key metabolites, which include BAs, SCFAs, and BCAAs (Figure 3).

The gut microbiota generates metabolites that affect host health by modulating the intestinal barrier, glucose homeostasis, the immune response, and lipid metabolism at the cellular, tissue, and organ levels. Among these metabolites, butyrate, propionate, and acetate are the primary products of resistant proteins and dietary fiber fermentation by bacteria in the gut. These SCFAs account for the majority of anions in the colon (Portincasa et al., 2022). They could meet 10% of the daily energy needs at the individual level in the human body, providing energy to body parts such as the gut, liver, and peripheral tissues. *Akkermansia muciniphila*, *Bacteroides*, *Bifidobacterium*, and *Clostridium* produce SCFAs primarily (Akhtar et al., 2022). They have advantageous effects in many biological processes within the system, comprising anti-inflammatory, immunomodulatory, anti-obesity, anti-diabetic, and anti-cancer actions, as well as providing cardiovascular protective, hepatoprotective, and neuroprotective benefits (Xiong et al., 2022).

Moreover, cholesterol metabolism is also regulated by the gut microbiome. Primary bile acids, which are synthesized in the liver and secreted into the intestine, are fermented by gut bacteria (e.g., *Lactobacillus* and *Clostridium*) to generate secondary bile acids [e.g., deoxycholic acid (DCA) and lithocholic acid (LCA)]. In addition, secondary bile acids not only influence the growth of gut microbiota in relation to bile acid metabolism but also modulate host metabolic processes by receptor activation, including the farnesoid X receptor (FXR) and G protein-coupled receptors (GPR) (Zhang Q. et al., 2023; Sanz et al., 2025).

Lipid metabolism disorders are a common pathological basis for metabolic diseases, and MFH substances targeting the gut microbiome have become a novel and highly promising therapeutic strategy for modulating lipid metabolism.

## 4 Interactions of gut microbiota and natural compounds from MFH substances in regulating lipid metabolic disorders

**FIGURE 4:**
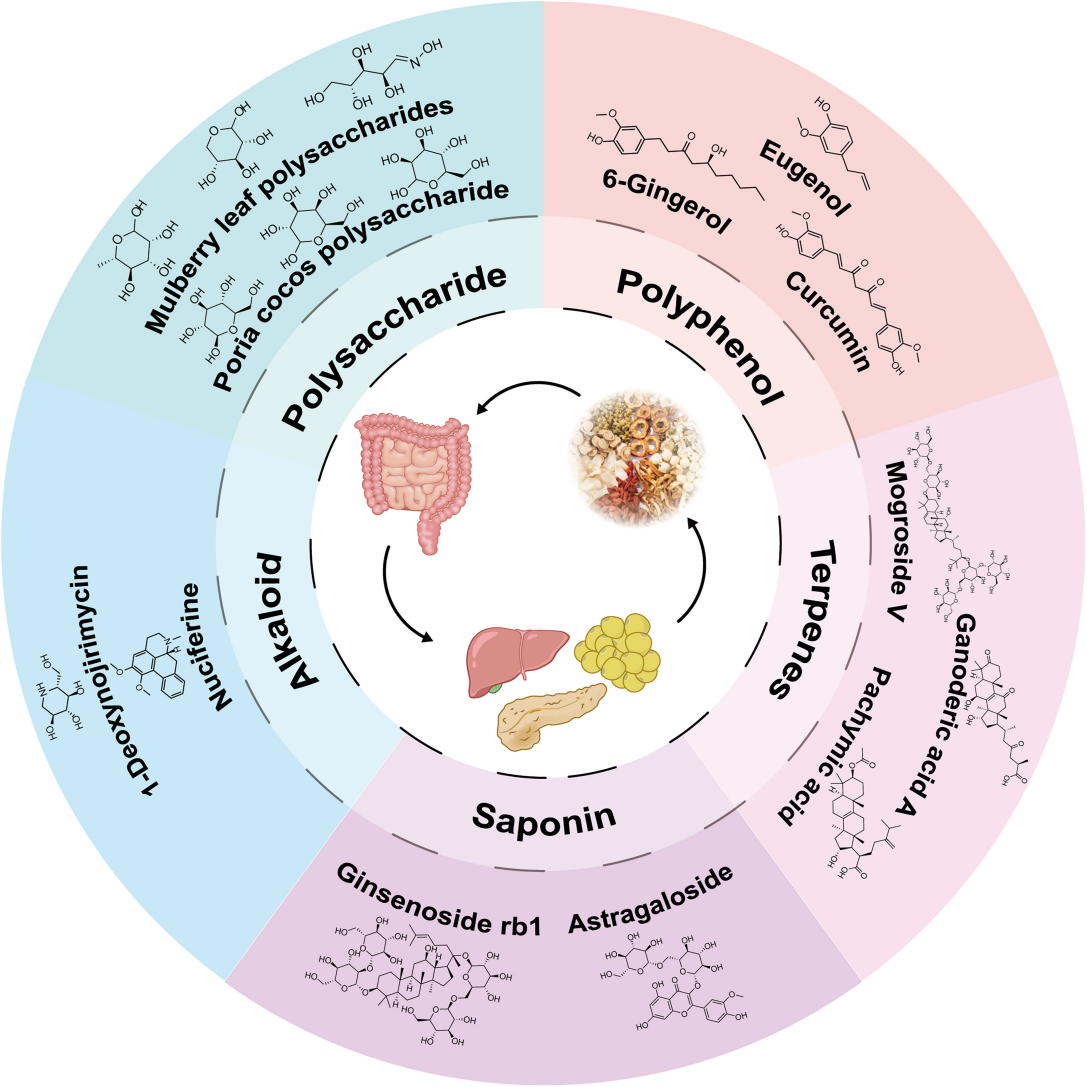
Natural compound classification and chemical structures in MFH substances.

Natural products are derived from plants, animals, bacteria, fungi, and marine sources. They are key to organisms’ defense mechanisms against stress and infections, making them a possible source of novel medications. The proportion of natural products and their derivatives among accepted drugs is substantial, and most of them pass clinical trials (Domingo-Fernández et al., 2024). Such examples are quinine, paclitaxel, vincristine, metformin, morphine, and aspirin, which have significantly increased and extended human health and life span. We group these compounds according to the chemical structures of the bioactive components of MFH, namely polysaccharides, polyphenols, saponins, alkaloids, and terpenoids (Figure 4).

### 4.1 Polysaccharides

Polysaccharides are high-molecular-weight polymers of monosaccharides linked by glycosidic bonds. They resist host digestion but are readily fermented by gut microbiota, generating bioactive metabolites that underlie their antioxidant, anticancer, antiviral, and immunomodulatory activities (Xue et al., 2025; Table 1).

**TABLE 1:** The role of polysaccharides targeting gut microbiota in lipid metabolic disorders.

Ingredients	Source	Disease	Experimental model	Dose and duration	Study types and sequencing method	Mechanism	Microbiota findings	References
Angelica sinensis polysaccharide	Angelica sinensis	NAFLD	*In vivo*: HFD-induced female BALB/c mice *In vitro*: AML12	*In vivo*: 160 mg/kg; 12 weeks *In vitro*: 500 μg/mL; 24 h	*In vivo*, 16S rRNA sequencing analysis; *In vitro*	↑HDL-C, ↓TG, LDL-C ↑CPT1A, ↓SCD1, GPAT4 ↑propionate, butyrate, ERRα Via propionate/ERRα pathway	*↑Akkemansia muciniphila, Clostridium leptum, Ruminococcus gnavus*	(Luo et al., 2023)
Chinese yam polysaccharides	Chinese yam	Inflammation	LPS-induced male C57BL/6J mice	100 mg/kg/day; 14 days	*In vivo*, 16S rRNA sequencing analysis	↓TNF-α, IL-6, IL-1β	*↑Prevotella* *↓Desulfovibrio*, *Sutterella*	(Wu S. et al., 2023)
Dioscorea opposita Thunb. polysaccharides	Dioscorea opposita Thunb.	Obesity	HFD-induced male C57BL/6J mice	60 mg/kg/day; 12 weeks	*In vivo*, 16S rRNA sequencing analysis	↓Body weight, insulin resistance ↓inflammation (TNF-α, IL-1β)	*↑Akkermansia* *↓Ruminiclostridium_9*	(Li S. N. et al., 2023)
Almond polysaccharide	Almond	Inflammation	LPS-induced RAW264.7 cells	100, 200, 500 μg/mL; 20 h	*In vitro*, 16S rRNA sequencing analysis	↑SCFAs (acetic acid, butyric acid) ↓NO, ROS ↓TNF-α, IL-1β, IL-6, iNOS	24 h: ↑*Clostridium*, *Megamonas* 48 h: ↑*Lactobacillaceae*, *Bifidobacteriaceae*	(Peng et al., 2024)
Raspberry polysaccharides	Raspberry	Obesity and inflammation	*In vivo*: HFD-induced male C57BL/6J mice *In vitro*: Caco-2 cells	*In vivo*: 100, 400 mg/kg/day; 12 weeks *In vitro*: 20, 40, 80μM; 24, 48 h	*In vivo*, 16S rRNA sequencing analysis; *In vitro*	↑HDL-C,↓TG, TC, LDL-C ↓hepatic steatosis ↓LPO, TNF-α, IL-6, IL-1β ↑ZO-1, Occludin, Claudin-5. Via TLR4/NF-κB signaling pathway	↑*Ruminococcaceae_UCG - 014, Lactobacillus taiwanensis, Bifidobacterium pseudolongum, Turicibacter*	(Huang et al., 2024)
Raspberry polysaccharides	Raspberry	Obesity-related liver injury	HFD-induced male C57BL/6J mice	100 mg/kg/day; 14 weeks	*In vivo*, FMT, metabolomics analysis, 16S rRNA sequencing analysis	↓Body weight, fatty liver ↑insulin sensitivity ↑HDL-C,↓TG, TC, LDL-C ↓IL-1β, IL-6, CYP2E1, NOX2, ACO, MDA ↑SOD, GSH, GCL-C, NQO1 ↑ZO-1 and Occludin ↑butyrate	↑*Dubosiella, Blautia, Acetatifactor*	(Wu et al., 2024)
Portulaca oleracea polysaccharides (POP)	Portulaca oleracea	Metabolic diseases	Aging SD male rats	1.0% POPs added to 99.0% pellet feed; 15 weeks	*In vivo*, 16S rRNA sequencing analysis, metabolomics analysis	↓TG, LDL-C, ALT, γ-GT ↓5β-cholestane-3α, 7α, 12α, 25-tetrol, vaccenic acid ↑soyasapogenol E, MG	↓The ratio of *Firmicutes/Bacteroidetes* ↓*Fusobacteria, Lactobacillus, Muribaculaceae*	(Fu et al., 2022)
Mulberry leaf polysaccharide	Mulberry leaf	Obesity	HFD-induced male C57BL/6N mice	50, 100 mg/kg/day; 20 weeks	*In vivo*, 16S rRNA sequencing analysis, plasma lipidomics analysis	↓Body, liver, adipose tissue weight ↑insulin sensitivity ↓the pathological lesions in colon	↓The ratio of *Firmicutes/Bacteroidetes* ↑*Muribaculum, Akkermansia, Anaeroplasma* ↓*Lactococcus, Mucispirillum, Desulfovibrio, Faecalibaculum*	(Zhao et al., 2022)
Mulberry leaf polysaccharides	Mulberry leaf	Obesity	HFD-induced male C57BL/6J mice	200, 400, 800 mg/kg/day; 8 weeks	*In vivo*, 16S rRNA sequencing analysis	↓Body weight, tissue weight ↓ALT, AST, ALP, LDH ↑HDL-C, ↓TG, TC, LDL-C ↓hepatic steatosis ↑UCP-1, PGC1-α ↑SCFA	↓The ratio of *Firmicutes/Bacteroidetes* ↑*Bifidobacterium, Lactobacillus, Akkermansia* ↓*Clostridiaceae, Desulfovibrio, Enterococcus*	(Li R. et al., 2022)
Mulberry leaf polysaccharides	Mulberry leaf	T2DM	HSHFD and STZ-induced SD male rats	100, 200, 400 mg/kg/day; 15 weeks	*In vivo*, 16S rRNA sequencing analysis	↓Blood glucose ↑insulin sensitivity, GLP-1 ↑HDL-C,↓TG, TC, LDL-C ↑fecal TBA,↓serum TBA, AST, ALT ↑Cyp7a1, Cyp8b1, Tgr5,↓Fxr Via the gut microbiota-bile acids metabolic pathway	↑*Prevotella, Ruminococcus, Lactobacillus*	(Dai et al., 2024)
Morus alba L. fruit polysaccharides	Morus alba L. fruit	MetS	HFD-induced male C57BL/6J mice	600 mg/kg/day; 14 weeks	*In vivo*, 16S rRNA sequencing analysis, untargeted fecal metabolomics analysis	↓Body weight, fat accumulation ↑SOD ↑insulin sensitivity ↓TG, TC, LDL-C ↓TNF-α, IL-1β, IL-6	↑*Muribaculum, Lachnospiraceae_NK4A136_group* ↓*Prevotella 2, Bacteroides, Faecalibacterium, Fusobacterium*	(Wan M. et al., 2022)
Pericarpium citri reticulatae polysaccharide	Pericarpium citri reticulatae	Obesity	HFD-induced male C57BL/6J mice	60 mg/kg/day; 4 weeks	*In vivo*, 16S rRNA sequencing analysis	↓Body weight, fat accumulation ↓TG, TC, LDL-C, ALT, AST Via the de novo synthesis and β-oxidation of fatty acid	↑*Lactobacillus johnsonii, Lachnospiraceae_NK4A136_group* ↓*Coriobacteriaceae_UCG_002*	(Li Y. et al., 2023)
Polygonatum rhizoma polysaccharide	Polygonatumrhizoma	T2D	Male db/db mice, m/m mice	1.0 g/kg/day; 6 weeks	*In vivo*, 16S rRNA sequencing analysis	↑Serum insulin, leptin, hepatic glycogen storage ↓PCK1 ↓NEFA, MDA, ↑SOD, GSH-Px, CAT	↑T*uricibacter genus* ↓*Lachnospiraceae, Romboutsia*	(Chen et al., 2023b)
Polygonatum polysaccharides	Polygonatum	Obesity	HFD-induced male C57BL/6J mice	60, 90, 130 mg/mL/day; 12 weeks	*In vivo*, 16S rRNA sequencing analysis	↓Fat accumulation ↑HDL-C, ↓TC, TG, NEFA, LDL-C, AST ↓HOMA-IR ↓IL-6,IL-1β,TNF-α ↓leptin, ghrelin, neuropeptide Y ↑GSH, CAT, SOD, T-AOC,↓MDA ↓SREBP-1c, Fas, ACC,↑AMPK ↑SCFAs (isobutyric acid, acetic acid, butyric acid) Via AMPK signaling pathways	↑*Lactobacillus* ↓*Dubosiella*	(Li L. et al., 2024)
Polygonatum kingianum polysaccharides	Polygonatum kingianum	MetS	HFD-induced SD rats	120, 240, 480 mg/kg/day; 14 weeks	*In vivo*, 16S rRNA sequencing analysis	↑HDL-C, ↓TG, TC, LDL-C, ALT, AST ↑PPARγ ↑SCFA ↑ZO-1, occludin ↓TLR4, IκB-α, IL-1β,↑IL-10 Via LPS-TLR4/NFκB signaling pathway	↑*Allobaculum, Phascolarctobacterium* ↓*Psychrobacter*	(Gu et al., 2020)
Radix puerariae thomsonii polysaccharide (RPP-2)	Radix puerariae thomsonii	NAFLD	HFD-induced male C57/BL6J mice	50, 100 mg/kg/day; 16 weeks	*In vivo*, 16S rRNA sequencing analysis, metabolomic analysis	↓Body weight, liver injury ↑butyric acid and propionic acid ↓D-mannitol ↑TJ proteins (ZO-1, Ocln, Cldn4), mucoprotein (MUC2) ↓LPS, TNF-α, Th17/Treg Via NF-κB and PPAR signaling pathway	↑*Butyricicoccus, Flintibacter, Oscillibacter* ↓the ratio of *Firmicutes/Bacteroidetes*	(Li Y. et al., 2023)
Astragalus mongholicus polysaccharides	Astragalus mongholicus	NAFLD	HFD-induced male SD rats	200 mg/kg/day; 10 weeks	*In vivo*, 16S rRNA sequencing analysis	↓Body weight ↓HOMA-IR ↑HDL-C,↓TC, TG, LDL-C, ALT, AST ↑AMPK, PPAR-α,↓SREBP-1, ↓TNF-α, TLR4, NF-κB, NLRP3 ↑ZO-1, occludin ↓GPR41, GPR43 Via LPS-TLR4-NF-κB-NLRP3 and AMPK-PPAR signaling pathway	↑*Proteobacteria, Epsilonbacteria* ↓the ratio of *Firmicutes/Bacteroidetes*	(Zhong et al., 2022)
Astragalus membranaceus polysaccharides	Astragalus membranaceus	T2DM	Male db/db mice	600 mg/kg/day; 16 days	*In vivo*, 16S rRNA sequencing analysis	↓FBG, HOMA-IR ↑GLP-1, GPR41, GPR43 ↑ZO-1, occludin ↑SCFAs	↑*Akkermansia, Faecalibaculum, Romboutsia*	(Chen X. et al., 2022)
Astragalus membranaceus polysaccharides	Astragalus membranaceus	T2DM	Male db/db mice and C57BL/6J mice	600 mg/kg/day; 16 days	*In vivo*, 16S rRNA sequencing analysis	↑GLP-1, GPCR41/43 ↓IL-6,↑TJ proteins (Occludin, ZO-1) ↑SCFA	↑*Bifidobacterium, Faecalibaculum, Akkermansia, Romboutsia*	(Song et al., 2022)
Astragalus membranaceus polysaccharides	Astragalus membranaceus	Hyperglycemia	LPS induced Caco-2 cell model; NCI-H716 cell	AMP:0.3125–10 mg/mL; SCFA sodium salt:1–64 mM	*In vitro*	↑Acetic acid, butyric acid, total SCFA ↑TJ proteins (ZO-1, occludin, Claudin-1) ↑GLP-1, GPCR43	↑*Dubosiella, Monoglobus* ↓*Escherichia-Shigella, Acinetobacter*	(Song et al., 2024)
Astragalus mongholicus polysaccharides	Astragalus mongholicus	T2DM	HSHFD and STZ-induced male	200 mg/kg/day; 8 weeks	*In vivo*, 16S rRNA sequencing analysis	↓FBG, HOMA-IR, TC, AST ↑ZO-1, occludin ↑PI3K, AKT, GLUT4 ↓IL-1β, TNF-α, TLR4, IκB, NF-κB Via PI3K/AKT and TLR4/NF-kB signaling pathway	↑*Ruminococcaceae, Bacteroides, Lachnospiraceae*, *Muribaculaceae* ↓*Proteobacteria* ↓the ratio of *Firmicutes/Bacteroidetes*	(Yuan et al., 2025)
Astragalus polysaccharide	Astragalus	T2DM	Human patients	*In vitro* fermentation model	*In vitro*, 16S sequencing analysis, metabolomics analysis	↑Acetic, butyric, propionic acid ↑all-trans-retinoic acid, thiamine, glutamine ↑antioxidant activity	↑*Lactobacillus, Bifidobacterium* ↓*Escherichia-Shigella*	(Zhang C. et al., 2024)
Astragalus polysaccharides (APS)	Astragalus	Metabolic disorders	HFD-induced male C57BL/6J mice	2, 4, 8% APS added in diet; 14 weeks	*In vivo*, 16S rRNA sequencing analysis, metabolomics analysis	↓Body weight, liver injury ↓TC, ALT, AST, FBG, INS Via purine metabolism pathway and glutathione metabolism pathway	↑*Bacteroidetes* ↓*Firmicutes, Deferribacteres*	(Hong et al., 2020)
Astragalus polysaccharides (APS)	Astragalus	NAFLD	HFD-induced male C57BL/6J mice	4% APS added in diet; 20 weeks	*In vivo*, 16S rRNA sequencing analysis, transcriptomics analysis	↓Serum INS ↑acetic acid ↑CPT1-α, PPARα ↓GCK, CD36, FASN	↑*Desulfovibrio vulgaris, Bacteroidetes* ↓*Firmicutes* ↓the ratio of *Firmicutes/Bacteroidetes*	(Hong et al., 2021)
Lotus seed resistant starch	Lotus seed	Hyperlipidemia	HFD-induced Kunming male mice	0.15, 0.3, 0.6 g/day/100 g; 8 weeks	*In vivo*, 16S rRNA sequencing analysis, metabolomics analysis	↑CA, formic acid ↓deoxycholic acid ↓medium chain fatty acids	↑*Muribaculacea, Erysipelotrichaceae* ↓*Allobaculum* ↓the ratio of *Firmicutes/Bacteroidetes*	(Zeng et al., 2023)
Lotus seed resistant starch	Lotus seed	Hyperlipidemia	HFD-induced SD male rats	/	*In vivo*, 16S rRNA sequencing analysis, metabolomics analysis	↑Primary, β-MCA, secondary bile acids (LCA, UDCA) ↓TCA, Dehydro-LCA, isoLCA, LCA-3-S, THDCA	↓*Romboutsia, Bacillus, Blautia, norank_f_Muribaculacea, norank_f_Eubacterium_coprostanoligenes_group*	(Lei et al., 2023)
Coix seed polysaccharides	Coix seed	T2DM	HFD and STZ-induced male C57BL/6J mice	175, 350 mg/kg/day; 9 weeks	*In vivo*, 16S rRNA sequencing analysis, colonic transcriptome analysis	↑HDL-C, ↓TG, TC, LDL-C ↑SCFA ↑ZO-1 ↑IGF1, AKT Via IGF1/PI3K/AKT signaling pathway	↑*Lactobacillus, Akkermansia, Bacteroides, Bifidobacterium* ↓*Helicobacter* ↓the ratio of *Firmicutes/Bacteroidetes*	(Xia et al., 2021)
Kelp resistant starch (KRS)	Kelp	Hyperglycemia	Hybrid snakeheads	10, 15% KRS added in diet; 60 days	*In vivo*, 16S rRNA sequencing analysis, metabolomic analysis	↓serum Glu, TC, TG ↑GSH-Px, SOD, CAT,↓MDA ↑α-Chmo, Lip, AKP, MCT1 ↓α-Ams	↑*Bacteroidetes, Actinobacteria* ↓*Firmicutes*	(Wang S. et al., 2023)
Insoluble dietary fibers (IDFs)	Laminaria japonica	Obesity	HFD-induced male C57BL/6 mice	2.5, 5% IDFs added in diet; 8 weeks	*In vivo*, 16S rRNA sequencing analysis	↑HDL-C,↓TG, TC, LDL-C, ALT, AST ↓SREBP-1c, Fas ↑acetate, propionate Via SREBP-1c/FAS signaling pathway	↑*Akkermansia*	(Zhang et al., 2021b)
Low-molecular alginate (LJA)	Laminaria japonica	Obesity and MS	HFD-induced male BALB/c mice	0.3% LJA added in drinking water; 11 weeks	*In vivo*, 16S rRNA sequencing analysis, FMT	↑HDL-C,↓TG, TC, LDL-C ↑CAT, SOD ↑acetate, propionate, butyrate ↓PPAR-γ,↑GPR41, GPR43, CPT-1A	↑*Bacteroidales* ↓*Clostridiales*	(Zheng et al., 2021)
Ginger polysaccharides	Ginger	Hyperlipidemia	HFD-induced male SD rats	50, 100, 200 mg/kg/day; 10 weeks	*In vivo*, 16S rRNA sequencing analysis	↓TG, TC, LDL-C ↑SOD, GSH-Px, T-AOC ↓TNF-α	↑*Akkermansia muciniphila* ↓the ratio of *Firmicutes/Bacteroidetes*	(Wu Q. H. et al., 2023)
Lycium barbarum polysaccharides-4	Lycium barbarum	T2DM	HFD-induced male C57BL/6J mice	200 mg/kg/day; 14 weeks	*In vivo*, 16S rRNA sequencing analysis, FMT	↓FBG, HOMA-IR ↑ZO-1, Claudin-1, MUC2 ↑GLP-1,↓LPS, IL-1β, IL-6 ↑SCFAs	↑*Allobaculum, Romboutsia*	(Zhou X. et al., 2023)
Lycium barbarum polysaccharides	Lycium barbarum	Obesity	HFD-induced male C57BL/6 mice	50 mg/kg/day; 12 weeks	*In vivo*, 16S rRNA sequencing analysis	↑HDL-C,↓TG, TC, LDL-C ↑SCFAs (acetic acid, propionic acid, butyric acid)	↑*Lactobacillus* ↓*Proteobacteria* ↓the ratio of *Firmicutes/Bacteroidetes*	(Yang Y. et al., 2021)
Lycium barbarum polysaccharides (LBPs)	Lycium barbarum	Obesity	HFD-induced male ICR mice	0.2% LBPs added in drinking water; 10 weeks	*In vivo*, 16S rRNA sequencing analysis	↑HDL-C,↓TG, TC, LDL-C ↓ACC1, FAS, SCD1, SREBP-1c, PPARγ, C/EBPα ↑butyric acid	↑*Lacticigenium, Lachnospiraceae_NK4A136_group, Butyricicoccus, Marvinbryantia* ↓the ratio of *Firmicutes/Bacteroidetes*	(Yang Y. et al., 2021)
Lycium barbarum polysaccharides	Lycium barbarum	T2DM	HFD and STZ-induced male C57BL/6 mice	50, 100, 200 mg/kg/day; 12 weeks	*In vivo*, 16S rRNA sequencing analysis	↓FBG, HbA1c, GSP, HOMA-IR ↑CAT, SOD, GSH-Px, T-AOC,↓MDA ↓IL-6, L-1β, TNF-α, LPS ↑SCFAs (acetate, propionate, butyrate) ↑InsR, IRS-1, IRS-2, PI3K, Akt, GLUT2 ↑GPR41, GPR43, GLP-1, PYY ↓GSK-3β, PEPCK Via IRS/PI3K/Akt signaling pathway	↑*Bacteroides, Ruminococcaceae_UCG-014, Intestinimonas, Mucispirillum, Ruminococcaceae_UCG-009* ↓*Allobaculum, Dubosiella, Romboutsia*	(Ma Q. et al., 2022)
Lycium barbarum polysaccharides	Lycium barbarum	DM	*In vivo*: HFD and STZ-induced male C57BL/6 mice; *In vitro*: Caco-2/RAW264.7 cells	100, 200 mg/kg/day; 12 weeks	*In vivo*, FMT, 16S rRNA sequencing analysis; *In vitro*	↓FBG, HbA1C ↓LPS, TNF-α, IL-6, IFN-γ ↑ZO-1, Claudin-1, Claudin-4 ↑butyrate	↑*Allobaculum (OTU_5)*	(Zhou et al., 2022)
Lycium barbarum polysaccharides	Lycium barbarum	Prediabetes	HFD-induced male C57BL/6J mice	50, 100, 150 mg/kg/day; 12 weeks	*In vivo*, 16S rRNA sequencing analysis	↑HDL-C,↓FBG, TC, TG, LDL-C ↑acetic acid	↑*Akkermansia, Lachnospiraceae_NK4A136_group*	(Li D. et al., 2023)
Lycium barbarum polysaccharides	Lycium barbarum	Hyperlipidemia/ NAFLD	Male Kunming mice	100, 200 mg/kg/day; 28 days	*In vivo*, 16S rRNA sequencing analysis, serum metabolomics analysis	↓TG, TC, LDL-C ↑T-AOC, SOD, ↓MDA ↑SCFAs ↑PPAR↑, ACOX1, CD36 Via PPARα signaling pathway	↑*Lactococcus*	(Liang et al., 2023)
Lycium barbarum polysaccharides	Lycium barbarum	NAFLD	HFD-induced male SD rats	50 mg/kg/day; 18 weeks	*In vivo*, 16S rRNA sequencing analysis	↑HDL-C,↓TC, TG, LDL-C, Leptin, FFA ↓FBG, serum INS, HOMA-IR ↑acetic acid, butyric acid, valeric acid ↑TJ (ZO-1, occludin),↓LPS, D-Lactate ↓TLR4, IKK, MyD88, p38MAPK, IκB, NF-κB p65 ↑IL-10, ↓IL-6, IL-1β,TNF-α,MCP-1 Via LPS/TLR4/NF-κB signaling pathway	↑*Deferribacteres* ↓*Verrucomicrobia, Enterococcaceae* ↓the ratio of *Firmicutes/Bacteroidetes*	(Gao et al., 2021)
Lycium barbarum polysaccharides	Lycium barbarum	T2DM	HFD and STZ-induced male SD rats	400 mg/kg/day; 12 weeks	*In vivo*, 16S rRNA sequencing analysis	↓LPS ↑IL-10,↓IL-1β,IL-6,IL-17A,TNF-α ↑acetic acid, valeric ↑TLR2γδ IELs, γδ IILs, ZO-1 (TJ, occludin) Via TLR2 signaling pathway	↑*Bifidobacterium, Lactobacillus, Alistipes* ↓*Desulfovibrio*	(Lu et al., 2021)
Poria cocos polysaccharides	Poria cocos	Obesity	HFD-induced male C57BL/6J mice	100 mg/kg/day; 8 weeks	*In vivo*, 16S rRNA sequencing analysis	↓CD36, FABP4, FATP2, FATP4 ↓TNF-α, VEGFA, TRL4 Via lipid and amino acid metabolism-related signaling pathways	↑*Bacteroidota* ↓*Actinobacteriota*	(Jiang S. et al., 2024)
Poria cocos polysaccharides	Poria cocos	Obesity	HFD-induced C57BL/6J mice	50 mg/kg/day; 13 weeks	*In vivo*, 16S rRNA sequencing analysis, FMT	↓Body weight, adipose tissue mass ↑SCFAs ↑FGF21, PI3K, AKT, GLUT4 Via FGF21/PI3K/AKT signaling pathway	↑*Lactobacillus, Allobaculum, Phascolarctobacterium*	(Liu W. et al., 2025)
Poria cocos polysaccharides	Poria cocos	NASH	MCD diet-induced C57BL/6J mice	150, 300 mg/kg/day; 4 weeks	*In vivo*, 16S rRNA sequencing analysis, transcriptomics analysis	↑HDL-C,↓ALT, AST ↑T-AOC,↓MDA, TNF-α, TLR4, NF-κB ↓CCR1, CCL3, CD11b Via NF-κB/CCL3/CCR1 signaling pathway	↑*Faecalibacterium*, *Escherichia_Shigella, Oscillospirales*	(Tan et al., 2022)
Carboxymethyl pachymaran	Poria cocos	Obesity	HFD-induced male C57BL/6J mice	400 mg/kg/day; 22 weeks	*In vivo*, 16S rRNA sequencing analysis, metabolomics analysis	↓TC, TG, LDL-C, ALT ↑butyric acid, isobutyric ↓bile acids (7-KDCA, CA-7S) Via cholesterol and lipid metabolism-related signaling pathways	↑*Akkermansia muciniphila, Bifidobacterium pseudolongum* ↓*Dubosiella newyorkensis, Faecalibacterium rodentium* ↓the ratio of *Firmicutes/Bacteroidetes*	(Gangzheng et al., 2023)
Ganoderma lucidum polysaccharides (F31)	Ganoderma lucidum	T2DM	HFD and STZ-induced male Kunming mice	60, 180 mg/kg/day; 9 weeks	*In vivo*, 16S rRNA sequencing analysis	↓FBG, HOMA-IR ↑HDL-C,↓TC, TG, LDL-C, ALT, AST ↑GSH-Px, SOD,↓MDA	↑*Lactobacillus, Bacteroides, Ruminococcaceae* ↓*Firmicutes*	(Shao et al., 2022)
Ganoderma lucidum polysaccharides	Ganoderma lucidum	Obesity	HFD-induced male C57BL/6J mice	100, 300 mg/kg/day; 12 weeks	*In vivo*, 16S rRNA sequencing analysis, FMT	↓eWAT, iWAT ↓IL-1β,IL-6,MCP-1 ↓CD11b, CD11c, CD68 ↑acetate, butyrate,↑GPR43 ↓LPS,↑TJ (occludin, Claudin-1, ZO-1) ↓CD14, TLR4, MyD88 Via TLR4/Myd88/NF-κB signaling pathway	↑*Allobaculum, Bifidobacterium* ↓*Lachnospiraceae_UCG-001, Ruminiclostrdium* ↓the ratio of *Firmicutes/Bacteroidetes*	(Sang et al., 2021)
Platycodon grandiflorus polysaccharides	Platycodon grandiflorus	Obesity	HFD-induced male C57BL/6J mice	500 mg/kg/day; 8 weeks	*In vivo*, 16S rRNA sequencing analysis, lipid metabolomics analysis, FMT	↓Body weight, fat accumulation ↑HDL-C,↓TC, TG, LDL-C ↑acetate, butyrate Via PPAR signaling pathway	↑*Muribaculaceae, Faecalibaculum*	(Ke et al., 2023)
Platycodon grandiflorus neutral polysaccharides	Platycodon grandiflorus	Obesity	HFD-induced male C57BL/6J mice	300 mg/kg/day; 14 weeks	*In vivo*, 16S rRNA sequencing analysis, fecal metabolomics analysis	↓TC, TG, LDL-C ↓IL-1β, TNF-α ↑ZO-1 ↑cholic, γ-linolenic acid Via primary bile acid biosynthesis and linoleic acid metabolism	↑*Bacteroides, Blautia* ↓*Rikenella, Helicobacter* ↓the ratio of *Firmicutes/Bacteroidetes*	(Song et al., 2023)
Platycodonis radix polysaccharides (PG1/PG2)	Platycodonis radix	Obesity	HFD-induced male C57BL/6J mice	0.1, 0.2, 0.4 g/kg/day; 90 days	*In vivo*, 16S rRNA sequencing analysis	↓LPS,↑TJ ↑SCFAs,↑GPR41, GPR43 ↑GLP-1, PYY ↓, FGFR4,↑CYP7A1 Via GM-SCFA-GPR and GM-BA-FXR-FGF15 signaling pathway	↑*Eubacterium coprostanoligenes, Lachnospiraceae_NK4A136, Akkermansia muciniphila* ↓the ratio of *Firmicutes/Bacteroidetes*	(Zhi et al., 2025)
Codonopsis pilosula polysaccharides	Codonopsis pilosula	liver injury	STC-induced male C57BL/6J mice	300 mg/kg/day; 2 weeks	*In vivo*, 16S rRNA sequencing analysis, liver transcriptome analysis, FMT	↓TC, TG, AST, ALT, LDH ↓TNF-α, IL-6, IL-1β, CCL2, CCL5 ↓CD36, CD68, MPO ↑SOD, CAT, GSH,↓MDA ↑Bcl-2,↓Bax, Caspase-3 ↑PPARα, Cpt1α ↑acetic acid	↑*Muribaculaceae, Lachnospiraceae, Oscillospiraceae, Alistipes* ↓*Helicobacter, Parasutterella, Escherichia_Shigella, Ligilactobacillus*	(Nie et al., 2024)
Codonopsis pilosula crude polysaccharides	Codonopsis pilosula	T2DM	HFD and STZ-induced male Wistar mice	1.0, 1.5, 2.0 g/kg/day; 4 weeks	*In vivo*, 16S rRNA sequencing analysis	↓TG, LDL-C, AST, ALT ↑SOD, GSH-Px, CAT, T-AOC,↓LPO, MDA ↓IL-6, NF-κB, TNF-α	↑*Bacteroides* ↓*Enterobacter* ↓the ratio of *Firmicutes/Bacteroidetes*	(Yang et al., 2023)
Oyster polysaccharides	Oyster	Obesity	HFD-induced male C57BL/6J mice	200, 600 mg/kg/day; 10 weeks	*In vivo*, 16S rRNA sequencing analysis	↑HDL-C,↓TC, TG, LDL-C ↓LPS, TNF-α, IL-1β, IL-6 ↑AMPKα,↓SREBP-1c, PPARγ, ACC-1 ↑acetic acid, propionic acid, butyric acid	↑*Dobosiella, Faecalibaculum* ↓*Erysipelatoclostridium, Mucispirillum*	(Ma Y. et al., 2022)
Dendrobium officinale polysaccharides	Dendrobium officinale	Obesity	*In vivo*: HFD-induced male SD rats; *In vitro*: PA-induced HepG2 liver cell	180 mg/kg/day; 90 days	*In vivo*, 16S rRNA sequencing analysis; *In vitro*	↑HDL-C,↓C-peptide, TC, TG, LDL-C ↓TNF-α, IL-6 ↑SOD,↓MDA ↓SOCS3,↑IRS-1 Via JAK/STAT/SOCS3 signaling pathway	↑*Muribaculaceae* ↓*Ralstonia, Pseudomonas*	(Jiang W. et al., 2024)
Dendrobium officinale polysaccharides	Dendrobium officinale	Prediabetic	HFHSD and STZ-induced male C57BL/6J mice	200 mg/kg/day; 6 weeks	*In vivo*, 16S rRNA sequencing analysis, FMT	↓TC, TG, AST ↓LPS, TNF-α, IL-6, ↓TLR4, IKKα, IκBα, NF-κB p65 ↑SCFAs, FFAR2/FFAR3, GLP-1, PYY Via SCFA-FFAR2/FFAR3 signaling pathway	↑*Bifidobacterium, Lactobacillus, Roseburia, Alloprevotella, Bacteroides* ↓*Colidextribacter, Helicobacter, Mucispirillum*	(Liu et al., 2023)
Dendrobium officinale polysaccharides	Dendrobium officinale	T2DM	HFD and STZ-induced male C57BL/6J mice	400 mg/kg/day; 12 weeks	*In vivo*, 16S rRNA sequencing analysis	↓FBG, FINS, HOMA-IR, TC, TG, LDL-C ↓TNF-α,IL-6,IL-1β ↑SOD, CAT, GSH,↓MDA ↑TJ (ZO-1, Occludin, Claudin-1) ↓LPS, TLR4, TRAM, TRIF, IKKβ, NF-κB p65 Via LPS/TLR4/TRIF/NF-κB signaling pathway	↑*Allobaculum, Bifidobacterium, Lactobacillus* ↓*Helicobacter*	(Chen et al., 2023a)
Dendrobium officinale polysaccharides	Dendrobium officinale	T2DM	STZ-induced male ICR mice	200, 400, 800 mg/kg/day; 5 weeks	*In vivo*	↓TC, TG, LDL-C ↑T-AOC, SOD, CAT ↑Glut2, Gck, Pklr, Insr, Pdx-1, Ins1 ↓Caspase-3	↑*Lactobacillus, Bifidobacteriaceae, Akkermansia*	(Li Y. et al., 2023)
Dendrobium officinale dietary fiber	Dendrobium officinale	Obesity	HFD-induced male C57BL/6J mice	1.0 g/kg/day; 11 weeks	*In vivo*, 16S rRNA sequencing analysis, metabolomics analysis, FMT	↓Body-weight, WAT, BAT ↑Gys2,↓Pck 1 ↓LPS, TNF-α, IL-1β ↑SOD,↓NOX2, NOX4 ↑acetate and taurine	↑*Akkermansia, Bifidobacterium, Muribaculum* ↓*Bilophila*	(Zheng et al., 2022)

#### 4.1.1 Astragalus

Astragalus is a dried root from the leguminous plant *Astragalus membranaceus (Fisch.) Bge. var. mongholicus (Bge.) Hsiao* or *Astragalus membranaceus (Fisch) Bge.* Astragalus, a typical medicinal herb of MFH, is widely utilized in China. Clinically, it is often employed for the management of diabetes, cancer, and cardiovascular and cerebrovascular diseases, with few adverse effects and high safety (Liu Y. X. et al., 2024). Astragalus polysaccharides (APS) are considered among the most pharmacologically active components of Astragalus. To date, approximately 30 different forms of APS have been detected and separated using different processing methods, including solvent, enzyme, and microwave-assisted extraction, for Astragalus (Tian et al., 2025). Glucans, neutral polysaccharides, acidic polysaccharides, and heteropolysaccharides are their primary constituents. These beneficial effects produced by APS were associated with the particular species of bacteria that were modulated and with improvements in bacterial metabolites.

APS treatment drastically enhanced the number of *Akkermansia*, *Faecalibaculum*, and *Romboutsia* in db/db mice. Maintaining structural integrity in the gastrointestinal tract, APS are selectively digested and consumed by intestinal microorganisms, thereby improving acetic acid (*p* < 0.001), propionic acid (*p* < 0.001), and butyric acid (*p* < 0.001) production. Such augmented SCFAs enhance intestinal barrier homeostasis by activating GPR41/43 (*p* < 0.001), which causes glucagon-like peptide-1 (GLP-1, *p* < 0.01) to be released and increases occludin (*p* < 0.001) and zonula occludens-1 (ZO-1, *p* < 0.05) (Song et al., 2022; Song et al., 2024). In T2DM rat models, APS reversed the *Firmicutes/Bacteroidetes* ratio and significantly reduced *Clostridia* and *Proteobacteria* (*p* < 0.05) (Yuan et al., 2025). *In vitro* research using simulated fermentation models demonstrated that APS efficiently remodel gut microbiota imbalance in people with T2DM by enriching *Bifidobacterium* and *Lactobacillus* while reducing *Escherichia-Shigella* (*p* < 0.01). Meanwhile, levels of L-glutamic acid, L-proline, L-valine, L-threonine, and spermidine (*p* < 0.05) were dramatically reduced by APS fermentation, whereas amounts of glutamine, thiamine, and all-trans retinoic acid (*p* < 0.05) were significantly increased (Zhang C. et al., 2024).

In C57BL/6J mice with metabolic illness brought on through a high-fat diet (HFD), APS lessened the quantity of *Firmicutes* and *Deferribacteres* (*p* < 0.01) while boosting the quantity of *Bacteroidetes* (*p* < 0.01) and then generating differential metabolites through the pathway of purine metabolism and glutathione metabolism, such as pyroglutamic acid, glutamic acid, uracil, inosine, deoxyguanosine, and guanosine (VIP > 1, *p* < 0.05). APS alleviated dyslipidemia by reducing fasting blood glucose (FBG, *p* < 0.01), insulin (*p* < 0.01) levels, total cholesterol (TC), aspartate aminotransferase (AST, *p* < 0.001), and alanine aminotransferase (ALT, *p* < 0.001) levels (Hong et al., 2020).

Furthermore, in an HFD-induced NAFLD mouse model, APS dramatically increased acetate levels (*p* < 0.05) and enhanced the abundance of *Desulfovibrio vulgaris* (*p* < 0.05), thereby suppressing hepatic expression of fatty acid synthase (FASN, *p* < 0.05) and CD36 protein (*p* < 0.05) expression. This indicates that APS may ameliorate hepatic steatosis via mechanisms involving *D. vulgaris*-mediated acetate production (Hong et al., 2021).

#### 4.1.2 Goji berry

Goji berries are dried, ripened fruits of *Lycium barbarum* L., which is a Solanaceae plant. Lycium polysaccharides (LBPs) are a complex of water-soluble sugars and represent the primary bioactive constituents of the goji berry. They have been extensively studied for their regulatory effects on metabolic health. LBPs are fermentation energy sources and fermentation substrates for gut microbes (Cao et al., 2022). LBPs are involved in the homeostasis of host health through changes in intestinal microecological balance, augmentation of bioactive metabolites, and gut-targeted interventions.

An obese mouse model induced by HFD was developed to investigate the anti-obesity properties of LBPs. The amount of *Lactobacillus*, *Lachnospiraceae_NK4A136_group*, and *Butyricicoccus* (*p* < 0.05) increased significantly following LBPs administration, and the amount of *Proteobacteria* (*p* < 0.05) declined. LBPs mitigated fat deposition in the liver and epididymis by downregulating key adipogenic genes, including acetyl-CoA carboxylase 1 (ACC1, *p* < 0.05), FASN (*p* < 0.05), stearoyl-CoA desaturase 1 (SCD1, *p* < 0.05), sterol regulatory element-binding protein-1c (SREBP1C, *p* < 0.05), peroxisome proliferator-activated receptor γ (PPARγ, *p* < 0.05), and CCAAT/enhancer-binding protein α (C/EBPα, *p* < 0.05). Therefore, LBPs improved hyperglycemia, hyperlipidemia, and disorders related to fat metabolism in mice (Yang Y. et al., 2021; Yang Y. et al., 2021).

LBPs-regulated glucose metabolism activated the signaling pathway IRS/PI3K/Akt in diabetic mice by altering the structure of the gut flora and enhancing the function of the intestinal barrier. LBPs intervention was observed to improve the counts for *Bacteroides*, *Mucispirillum*, *Intestinimonas*, and *Ruminococcaceae* (*p* < 0.05), at the expense of *Allobaculum*, *Dubosiella*, and *Romboutsia* (*p* < 0.05) (Ma Q. et al., 2022; Zhou et al., 2022). The intestinal barrier protection pathway enhances barrier function by increasing ZO-1 (*p* < 0.05) expression. This effect has been confirmed in diabetic mice and the models of the Caco-2/RAW264.7 cell co-culture models of inflammation (Zhou et al., 2022). Also, the gut-metabolic axis control pathway can augment duodenal contractile responses via microbiota-elicited mechanisms, leading to enhanced glucose homeostasis (Li D. et al., 2023).

NAFLD is significantly prevented by LBPs. LBPs intervention in NAFLD mice enhances the growth of *Lactobacillus*, *Lactococcus*, and *Bacteroidetes* (*p* < 0.05) and decreases the number of *Proteobacteria* (*p* = 0.01). In discovery, trichloroacetic acid and acetic acid (*p* < 0.05) were engaged in the enrichment of fatty acid degradation processes. Seven differential metabolites (VIP ≥ 1, *p* < 0.05), the levels of 4-chloro-2-methylphenol, PC[18:1(9Z)/18:1(9Z)], and PC[18:0/16:1(9Z)] were considerably upregulated, whereas trichloroacetic acid, acetic acid, melampodinin, and 8-desoxygartanin displayed a downward trend (Gao et al., 2021; Liang et al., 2023). All of these are protective actions, including maintaining the equilibrium of the gut microbiota homeostasis, repairing the intestinal epithelial barrier, blocking the entry of LPS and hepatic LPS-binding proteins into the bloodstream, suppressing hepatic inflammatory responses, and activating the fatty acid β-oxidation pathway. Lastly, it minimizes liver damage and fat buildup by coordinating the gut-liver axis.

#### 4.1.3 Poria

Poria, a sclerotium of the fungus *Poria cocos* (Schw.) Wolf has been used therapeutically for over 2,500 years. Poria cocos polysaccharides (PCPs), which are the principal constituent of 70–90% of the dry sclerotium mass (Ng et al., 2024), are mainly made up of mannose, glucose, galactose, fucose, and D-glucosamine hydrochloride (Duan et al., 2023). By the gut microbiota, PCPs are readily metabolized, producing significant microbial metabolites that regulate glycolipid metabolism and host health.

PCPs ameliorated the pathological alterations in colon tissues and reduced fatty acid transport genes, including CD36 (*p* < 0.01), fatty acid-binding protein 4 (FABP4, *p* < 0.01), fatty acid transport protein 2 (FATP2, *p* < 0.01), and fatty acid transport protein 4 (FATP4, *p* < 0.01), in obese mice. PCPs raised the abundance of bacterial taxa involved in lipid and amino acid breakdown, including *Eisenbergiella*, *Dorea*, *Proteiniphilum*, and *Lachnospira* (Jiang S. et al., 2024). It helps to minimize the intestinal lipid overload to increase the microenvironmental balance, which ultimately regulates the signaling pathways related to obesity.

Meanwhile, PCPs have a positive effect on the proportional increase in *Lactobacillus*, *Allobaculum*, and *Phascolarctobacterium*, thereby enhancing acetate (*p* < 0.01), propionate (*p* < 0.01), and butyrate (*p* < 0.05) production. It acts through activated fibroblast growth factor 21 (FGF21, *p* < 0.001), phosphoinositide 3-kinase (PI3K, *p* < 0.01), protein kinase B (AKT, *p* < 0.01), and glucose transporter 4 (GLUT4, *p* < 0.001) expression, reducing IR in fat by promoting glucose and lipid metabolism (Liu W. et al., 2025).

PCPs lowered liver tissue damage, liver dysfunction, and hepatic oxidative stress in methionine and choline deficient (MCD)-induced NASH mice in C57BL/6J. Its main activity is to increase the proportion of *Faecalibacterium* (*p* < 0.05) and lower the amount of endotoxins produced by gut bacteria. The expression levels of C-C motif chemokine ligand 3 (CCL3, *p* < 0.05), C-C motif chemokine receptor 1 (CCR1, *p* < 0.05), toll-like receptor 4 (TLR4, *p* < 0.05), cluster of differentiation 11b (CD11b, *p* < 0.05), nuclear factor kappa-B (NF-κB, *p* < 0.05) and tumor necrosis factor-α (TNF-α, *p* < 0.05) were reduced. By modulating the gut microbiota and the NF-κB/CCL3/CCR1 signaling axis, PCPs effectively halt NASH progression (Tan et al., 2022).

#### 4.1.4 Dendrobium officinale

The dried stem of the orchid *Dendrobium officinale Kimura et Migo* is known as Dendrobium officinale. Dendrobium officinale polysaccharides (DOPs) are the unique active constituents of the plant that are vital polysaccharides, which facilitate the functional effects of the plant. Their major compositions were glucose, mannose, xylose, and galactose (Guo et al., 2025). DOPs regulate gut microbiota diversity and composition, mediate systemic anti-inflammatory effects by altering metabolite levels, and support improvements in disease and physiological homeostasis.

In an obese rat, DOPs altered the gut microbial makeup by raising the quantity of *Muribaculaceae* while minimizing that of *Ralstonia* and *Pseudomonas*. DOPs-mediated microbial changes reduced hepatic damage and insulin resistance by inhibiting suppressor of cytokine signaling 3 (SOCS3, *p* < 0.001) and promoting insulin receptor substrate-1 (IRS-1, *p* < 0.001) expression. In addition, the efficacy of the DOPs intervention in treating obesity is demonstrated in a HepG2 hepatocyte IR model, in which it restored abnormal protein expression in the JAK2/STAT3/SOCS3 signaling pathway (Jiang W. et al., 2024).

Among diabetic mice, DOPs reduced the likelihood of progression from prediabetes to T2DM by 63.7%. *In vivo* and *in vitro* studies are unanimous that DOPs can remodel the gut microbiota by reducing *Helicobacter pylori* and increasing *Bifidobacterium*, *Lactobacillus*, and *Allobaculum*. LPS leakage, metabolic inflammation, and dysfunctional glucolipid metabolism were ameliorated by DOPs. DOPs reduced serum markers of intestinal mucosal injury, LPS (*p* < 0.05), D-lactic acid (D-LA, *p* < 0.05), and diamine oxidase (DAO, *p* < 0.05), and serum content of pro-inflammatory factors TNF-α (*p* < 0.001), interleukin-6 (IL-6, *p* < 0.001), and interleukin-1β (IL-1β, *p* < 0.001). Additionally, superoxide dismutase (SOD, *p* < 0.05), catalase (CAT, *p* < 0.05), and glutathione (GSH, *p* < 0.05) levels indicated a recovery trend. The expression of these key proteins, TLR4 (*p* < 0.001), TRIF-related adaptor molecule (TRAM, *p* < 0.001), and TIR-domain-containing adapter-inducing interferon-β (TRIF, *p* < 0.001), was significantly reduced (Chen et al., 2023a; Liu et al., 2023). The change of gut flora is essential for the aforementioned benefits associated with DOPs.

### 4.2 Polyphenols

Gut bacteria ferment polyphenols—such as flavonoids, lignans, and tannins—thereby affecting their bioavailability and activity (Matsumura et al., 2023). These compounds influence host health by reshaping gut microbial composition, altering enzyme activity, and changing metabolite profiles (Cheng et al., 2023; Table 2).

**TABLE 2:** The role of polyphenols targeting gut microbiota in lipid metabolic disorders.

Ingredients	Source	Disease	Experimental model	Dose and duration	Study types and sequencing method	Mechanism	Microbiota findings	References
Eugenol (EU)	Clove oil	Obesity	HDF-induced male C57BL/6J mice	0.2% EU added in diet; 13 weeks	*In vivo*, 16S rRNA sequencing analysis, transcriptome analysis	↓body weight, FBG ↑LHB, Adcy8, CFTR, Calm14, Tnni3, Gli1 Via cAMP signaling pathway	↑*Dubosiella, Blautia, Oscillospiraceae, Ruminococcaceae* ↓*Alistipes, Alloprevotella, Bilophila*	(Li R. et al., 2022)
Hawthorn procyanidins	Hawthorn	LMD	HDF-induced male Wistar rats	50, 100, 200 mg/kg/day; 16 weeks	*In vivo*, 16S rRNA sequencing analysis	↑HDL-C, ↓TC, TG, LDL-C, ALT, AST ↑adiponectin, ↓leptin ↑SOD, GSH, ↓MDA ↑SCFAs ↓LPS, TLR4, NF-κB, MyD88, IKKβ ↓IL-1β, TNF-α ↑ACC,↓SREBP-1c ↑occludin, claudin-1 Via NF-κB and AMPK signaling pathways	↑*Akkermansia, Bacteroides, Adlercreutzia* ↓*Lactobacillus, Bifidobacterium, Blautia, Lachnospiraceae, Subdoligranulum*	(Han et al., 2022)
Hawthorn total flavonoids	Hawthorn	hyperlipidemia	*In vivo*: HFD-induced male C57BL/6J mice; *In vitro*: HepG2 cells	*In vivo*: 0.025, 0.05 g/kg/day; 9 weeks *In vitro*: 150, 175, 200, 225, 250, 275μM	*In vivo*, 16S rRNA sequencing analysis; *In vitro*	↓Body weight, fat accumulation ↓AST, LDH ↑AMPK, ↓SREBP-1c, ACC, FASN, HMG-CoA ↑PPARα/γ, CPT-1A ↑Cholesterol 7α-hydroxylase	↓The ratio of *Firmicutes/Bacteroidetes*	(Zheng et al., 2025)
Mulberry polyphenols	Mulberry	T2DM	Male db/db mice and m/m mice	400, 900 mg/kg/day; 8 weeks	*In vivo*, 16S rRNA sequencing analysis	↓FBG, FIN ↑HDL-C,↓TC, LDL-C ↑CAT, GSH-Px, SOD,↓NO ↑GLUT-4 ↑propionate, butyrate	↑*Bacteroides* ↓*Firmicutes, Lactobacillus, Bacillus*	(Li and Ming, 2024)
Red raspberry polyphenolic (RR)	Raspberry	Obesity	HFD-induced male C57BL/6J mice	RR added in diet; 16 weeks	*In vivo*, 16S rRNA sequencing analysis	↓WAT, BAT	↑*Roseburia, Bifidobacterium* ↓*Mogibacteriaceae*	(Xian et al., 2021)
Pelargonidin-3-O-glucoside	Raspberry	T2DM	*In vivo*: db/db diabetic mice; *In vitro*: HepG2 cells model	*In vivo*: 150 mg/kg/day; 8 weeks *In vitro*: 2.5, 5, 10, 20μg/mL	*In vivo*; *In vitro*	↑LC3B, TFEB ↓FIN, HOMA-IR ↑SCFAs ↑occludin, ZO-1 ↑TLR2, Pla2g2, Lyz1	↑*Prevotella* ↓the ratio of *Firmicutes/Bacteroidetes*	(Su et al., 2020)
Rhododendron (RHO)	Raspberry	NAFLD	HFD-induced male C57BL/6J mice	0.2% RHO added in diet; 16 weeks	*In vivo*, 16S rRNA sequencing analysis, liver metabolomics analysis	↓TC, TG, LDL-C, ALT, AST ↓TNF-α, IL-1β, IL-6, MCP1 Via amino acid metabolism signaling pathways	↑*Oscillibacter, Lachnospiraceae_bacterium_28_4, Bacteroides sartorii* ↓the ratio of *Firmicutes/Bacteroidetes*	(Li X. et al., 2022)
Mulberry leaves flavonoids	Mulberry leaf	Obesity	HFD-induced female ICR mice	240 mg/kg/day; 6 weeks	*In vivo*, 16S rDNA sequencing analysis, FMT	↓Body weight, iWAT, eWAT, pWAT ↓ACC, PPARα/γ, SREBP1/2, LXRα/β ↑UCP1, CPT-1β, PGC-1α, FABP5 ↑acetic acid	↑*Bacteroidetes, Rickettsiales, Arenimonas, Clostridiales* ↓the ratio of *Firmicutes/Bacteroidetes*	(Zhong et al., 2020)
Morus alba L. fruit polyphenols	Morus alba L. fruit	MetS	HFD-induced male C57BL/6J mice	300 mg/kg/day; 14 weeks	*In vivo*, 16S rDNA sequencing analysis, fecal metabolomics analysis	↓Body weight, fat accumulation ↑SOD ↑insulin sensitivity ↓TG, TC, LDL-C ↓TNF-α, IL-1β, IL-6	↑*Muribaculum, Lachnospiraceae_NK4A136_group* ↓*Prevotella 2, Bacteroides, Faecalibacterium, Fusobacterium*	(Wan M. et al., 2022)
Polymethoxyflavone	Aged citrus peel	Obesity	SHIME	15 Days	*In vitro*, 16S rRNA sequencing analysis	↓Acetic, propionic, butyric acid	↑*Roseburia, Blautia, Subdoligranulum, Eubacterium*	(Falduto et al., 2022)
Miquelianin (quercetin 3-O-glucuronide, Q3G)	Folium nelumbinis	Obesity	*In vivo*: HFD-induced male C57BL/6J mice; *In vitro*: 3T3-L1 adipocytes	*In vivo*: 12.8, 25.6 mg/kg/day; 12 weeks *In vitro*: 100, 200 μg/mL	*In vivo*, 16S rRNA sequencing analysis; *In vitro*	↓Body, WAT ↑HDL-C,↓TC, TG, LDL-C ↑SIRT1, COX2, PPARGC-1α, TFAM, UCP1 ↓PINK1, Parkin, BECLIN1, LC-3B Via AMPK/DRP1/mitophagy signaling pathway	↑*Mucispirillum* ↓*Faecalibaculum, Colidextribacter*	(Wang S. et al., 2023)
Coix seed polyphenols	Coix seed	Obesity	MKN28, Caco-2, 3T3-L1 cell	100, 200 μg/mL	*In vitro*	↓TC, TG, LDL-C ↓ROS	↑*Lactobacillus, Bifidobacterium*	(Qin et al., 2023)
6-Gingerol	Ginger	Obesity	HFD-induced male C57BL/6J mice	50 mg/kg/day; 16 weeks	*In vivo*, 16S rRNA sequencing analysis, metabolomics analysis, FMT	↓iWAT, eWAT, pWAT ↑HDL-C, ↓TC, TG, LDL-C, ALT, AST Via ABC transporters, arginine biosynthesis, and arginine and proline metabolism signaling pathways	↑*Muribaculaceae, Alloprevotella, Akkermansia* ↓*Lachnospiraceae, Lactobacillus reuteri*	(Yasmin et al., 2023)
Zingerone (ZIN)	Ginger	Obesity	HFD-induced male C57BL/6J mice	0.2% ZIN added in diet; 16 weeks	*In vivo*, 16S rRNA sequencing analysis	↓Body weight, iWAT, eWAT ↓TC, TG, LDL-C, ALT, AST ↑UCP1, PGC-1α, PRDM16 ↑UCP2, CPT1β, ADRB3, ELOVL3, PPARα Via PPARα signaling pathways	↑*Akkermansia_mucinphila* ↓the ratio of *Firmicutes/Bacteroidetes*	(Li D. et al., 2023)
Amomum tsao-ko polyphenols	Amomum tsao-ko	Hypercholesterolemia	HCD-induced male Golden Syrian hamsters	1,000 mg/kg/day; 6 weeks	*In vivo*, 16S rRNA sequencing analysis	↓TC, TG ↓atherosclerotic plaque area ↑fecal total acidic sterols ↑SCFAs ↑CYP7A1, LXRα Via bile acid metabolism signaling pathways	↑*Ruminococcus_2* ↓*Allobaculum, Desulfovibrio*	(Liu et al., 2021)
Curcumin (Cur)	Curcuma longa	Obesity	HFD-induced male C57BL/6J mice	0.4% Cur added in diet; 14 weeks	*In vivo*, 16S rRNA sequencing analysis	↑GLUT4 ↓M1 (Cd80, Cd38, Cd11c), M2 (Arginase-1) ↓NF-κB p65, STAT1, TLR4, IL-6	↑*Lactococcus, Parasutterella, Turicibacter*	(Islam et al., 2021)
Curcumin	Curcuma longa	Obesity	HFD-induced male C57BL/6J mice	50, 250, 500 mg/kg/day; 12 weeks	*In vivo*	↓TC ↑IL-10,↓TNF-α,NF-κB,MCP-1 ↓Arginase-1	↑*Clostridium clusters IV and XIVa*	(Bertoncini-Silva et al., 2023)
Curcumin (Cur)	Curcuma longa	Obesity	HFD-induced male C57BL/6J mice	0.2% Cur added in diet; 10 weeks	*In vivo*, 16S rRNA sequencing analysis	↓Body weight ↓TC, TG, ALT, AST ↓SREBP-1c, FAS, SCD1 ↑CPT-1α, PDK4, MCAD, PPARα ↓LPS ↑butyric acid, isobutyric acid	↑*Akkermansia, Alloprevotella* ↓the ratio of *Firmicutes/Bacteroidetes*	(Li et al., 2021)
Curcumin	Curcuma longa	Obesity	HFD-induced wild type gene knockout mice	100 mg/kg/day; 12 weeks	*In vivo*, 16S rRNA sequencing analysis, FMT	↑Ucp1 ↑β-MCA, DCA, LCA,↓TCA ↓Cyp7a1, Cyp8b1 ↑TGR5 ↑cAMP, CREB Via cAMP/PKA signaling pathway	↑*Lactobacillus, Clostridium cluster XIVa, Akkermansia*	(Han et al., 2021)
Curcumin	Curcuma longa	Obesity	male C57BL/6J HamSlc-ob/ob mice (ob/ob, C57BL/6J background)	60 mg/kg/day; 8 weeks	*In vivo*, 16S rRNA sequencing analysis	↓Body weight, iWAT, BAT ↓IL-1, IL-6, TNF-α ↑ZO-1, occludin ↑Cyp7a1, CYP7b1, Cyp27a1 ↑DCA, TGR5,↓FXR ↑GLP-1 Via the gut microbiota-BAs-TGR5/FXR axis	↓*Lactobacillus* ↓the ratio of *Firmicutes/Bacteroidetes*	(Tian et al., 2023)
Calebin-A	Curcuma longa	Obesity	HFD-induced male C57BL/6J mice	0.1 and 0.5% Calebin-A added in diet; 12 weeks	*In vivo*, 16S rRNA sequencing analysis	↓Body weight, FBG ↓WAT, BAT ↑rectal temperature	↑*Akkermansia, Butyricicoccus, Ruminiclostridium_9*	(Lee et al., 2023)
Curcumin	Curcuma longa	T2DM	HFD and STZ-induced male rats	200 mg/kg/day; 16 weeks	*In vivo*, 16S rRNA sequencing analysis, metabonomics analysis	↓DAO, ↑TJ (Occludin, ZO-1) ↓FBG, HOMA-IR ↓LPS, TNF-α ↓TLR4, NF-κB Via TLR4/NF-κB signaling pathway	↑*Bacteroidetes, Bifidobacterium* ↓*Enterobacterales, Firmicutes*	(Huang et al., 2021)
Curcumin (Cur)	Curcuma longa	NAFLD	BPA-induced male CD-1 mice	0.1% Cur added in diet; 24 weeks	*In vivo*, 16S rRNA sequencing analysis	↑HDL-C, ↓TC, TG, LDL-C, ALT, AST ↑TJ (Occludin, ZO-1) ↓LPS, DAO, D-lactate ↓TNF-α, IL-6, IL-1β, IL-18 Via TLR4/NF-κB signaling pathway	↑*Akkermansia* ↓the ratio of *Firmicutes/Bacteroidetes*	(Hong et al., 2022)
Curcumin	Curcuma longa	NAFLD	HFD-induced male SD rats	200 mg/kg/day; 14 weeks	*In vivo*, 16S rRNA sequencing analysis	↓TNF-α, IL-1β, IL-6, IL-23, IP-10, OX40	↑*Butyricicoccus* ↓*Dorea* ↓the ratio of *Firmicutes/Bacteroidetes*	(Li R. et al., 2020)
Tetrahydrocurcumin	Curcuma longa	T2DM	C57BL/6J mice, db/db mice	100,200 mg/kg/ day; 8 weeks	*In vivo*, 16S rRNA sequencing analysis	↓FBG ↑GLP-1	↓*Actinobacteria* ↓the ratio of *Firmicutes/Bacteroidetes*	(Yuan et al., 2020)
Lycium barbarum flavonoids	Lycium barbarum	T2DM	HFD and STZ-induced male C57BL/6J mice	100, 200 mg/kg/day; 12 weeks	*In vivo*, 16S rRNA sequencing analysis	↑Body weight ↓liver index, FBG, HOMA-IR, HOMA-IS, HbA1c, ↑GLP-1 ↓TC, TG ↓LPS, TLR-4, TNF-α, IL-6, IL-10, IFN-γ ↓G6Pase, PEPCK, PPARγ, FAS, ACC, SREBP-1c ↑GK, PPK, PPARα	↓*Lachnospiraceae, Lactobacillaceae* ↓the ratio of *Firmicutes/Bacteroidetes*	(Yang et al., 2022)
Sesamol	Sesame	Obesity	*In vivo*: DFO-induced male C57BL/6J mice; *In vitro*: Caco-2 cell	0.2% Sesamol added in diet; 8 weeks	*In vivo*, 16S rRNA sequencing analysis; *In vitro*	↓TPC, OX-TG ↑HDL-C, ↓TC ↑PPARα ↑Cpt1, Cpt2, Acot1, Acot2, MCAD ↑Mnsod, Cat,↓Acaca, Acsl3 ↓MDA, iNOS, IL1β ↑TJ (Claudin-1, Occludin, and ZO-1)	↑*Bifidobacterium, Akkermansia*	(Wang et al., 2022a)
Cornus tannin	Cornus fructus	T2DM	HFD and STZ-induced male ICR mice	400 mg/kg/day; 14 weeks	*In vivo*, 16S rRNA sequencing analysis	↑Body weight, ↓FBG, serum FINS ↑HDL-C, ↓TC, TG, LDL-C, ALT, AST ↑GLP-1, GLP-2, GPR43 ↑acetate, propionate, butyrate	↑*Lactobacillus, Clostridium*	(Niu et al., 2020)

#### 4.2.1 Raspberry

Raspberry, the dried fruit of the Rosaceae family, *Rubus chingii Hu*, is widely found throughout the world’s temperate regions. Raspberries contain high levels of physiologically active natural polyphenols, including ellagic acid (EA) and anthocyanins. A previous study found that most raspberry polyphenols are metabolized in the colon to EA and urolithin derivatives. These metabolites are absorbed through the intestine and, after interaction with the liver, bind to the primary molecular targets that regulate metabolic balance, inflammatory response, and oxidative stress (Fotschki et al., 2023).

The intervention with raspberry polyphenol (RR) in obese mice yielded positive results in increasing *Roseburia* and *Adlercreutzia* (*p* < 0.05) and decreasing *Ruminococcus_1* (*p* < 0.05) within the gut microbiota, in this case, synergistically alleviating effects on hepatic, brown adipose tissue (BAT), and pancreatic tissues’ TG deposition and inflammation (Xian et al., 2021).

A typical anthocyanin, Pelargonidin-3-O-glucoside (Pg3G), present in raspberry, has been shown to inhibit α-glucosidase activity in diabetic mice. It enriches *Prevotella* (*p* < 0.05) and the *Bacteroidetes/Firmicutes* ratio (*p* < 0.05), and by selectively up-regulating the antimicrobial peptide genes phospholipase A2 group-II (Pla2g2, *p* < *0.05*) and Lysosome-1 (Lyz1, *p* < 0.05), it improves intestinal barrier function. Proteins related to microtubules, light chain 3B (LC3B, *p* < 0.05) and transcriptional factor EB (TFEB, *p* < 0.05), are activated. Such modulation ultimately raises sensitivity to insulin, tolerance to glucose, and autophagy induction in T2DM animals and in high-glucose/high-fat (HG + HF)-induced hepatocytes (Su et al., 2020).

Rubusoholic acid (RHO), which is a primary metabolite of raspberry ketone (RK), can be used to improve NAFLD. RHO selectively enriched positive gut bacterial species, including *Bacteroides*, *Bilophila*, *Oscillibacter*, *Leptospiraceae_bacterium_28_4*, and *Barnesiella sartorii*, which led to significant weight (*p* < 0.05) and epididymal fat mass (*p* < 0.05) loss in the mice. It decreases TNF-α (*p* < 0.05), IL-1β (*p* < 0.05), monocyte chemoattractant protein 1 (MCP1, *p* < 0.05), IL-6 (*p* < 0.05), and ALT/AST (*p* < 0.05) levels. RHO alleviated hepatic inflammatory infiltration and fat accumulation. These metabolites, for example, L-(+)-aspartic acid, D-proline, and ornithine (VIP > 1, *p* < 0.05), were enriched after RHO supplementation. Its anti-obesity and hepatic protective activities are intimately associated with the gut-liver axis metabolic pathways via regulation of the alanine-aspartate-glutamate and arginine-proline metabolic pathways (Li X. et al., 2022).

#### 4.2.2 Mulberry leaf

The dried leaves of *Morus alba L.* are called the mulberry leaves. It contains several bioactive compounds, comprising polysaccharides, flavonoids, and alkaloids. Mulberry leaves reduce blood lipids, blood sugar, and inflammation, and increase antioxidants, which are beneficial for protecting pancreatic cells, alleviating IR, and working with gut flora (Zhang Q. et al., 2023). Numerous studies have shown that mulberry leaf flavonoid (MLF) can alleviate lipid metabolic diseases by controlling the gut flora.

Animal experiments and microbiota transplantation experiments revealed that administration of MLF improved lipid metabolism, mediated by *Bacteroidetes*-driven acetic acid production. In ICR mice, MLF intervention effectively reduced weight gain (*p* = 0.0895) and white adipose tissue (WAT) weights in inguinal (iWAT, *p* = 0.0607), epididymal (eWAT, *p* < 0.01), and perirenal (pWAT, *p* < 0.01) regions, as well as TG levels (*p* < 0.05). On the molecular level, MLF significantly suppressed the expression of lipogenic genes, including ACC (*p* < 0.05), peroxisome proliferator-activated receptor α (PPARα, *p* < 0.05), sterol regulatory element-binding protein 2 (SREBP2, *p* < 0.05), and SREBP1 (*p* = 0.0807). It enhanced the expression of thermogenic and fatty acid oxidation markers, such as uncoupling protein 1 (UCP1, *p* = 0.0653), carnitine palmitoyltransferase1β (CPT-1β, *p* = 0.0595), PPARγ coactivator 1-α (PGC-1α, *p* < 0.05), and fatty acid-binding protein 5 (FABP5, *p* < 0.05) (Zhong et al., 2020).

The higher levels of *Lactobacillus vaginalis* and *Lactobacillus gasseri* could lead to a problem with lipid metabolism. Mulberry leaf polyphenols (MLP) have anti-obesity effects by reducing *Firmicutes* (*p* < 0.05) abundance and the downstream *Clostridiales* and *Lachnespiraceae* (*p* < 0.05). Concurrently, *Lactobacillus vaginalis* and *Lactobacillus gasseri* species are declining. Moreover, MLP normalized the amino acid and oligopeptide metabolic disorder in obese rats, including indoles (2-indolecarboxylic acid, indoleacetic acid), histidine, urocanic acid, aspartyl-leucine, aspartyl-glutamic acid, and phenylalanyl-hydroxyproline (*p* < 0.05) (Li et al., 2019).

#### 4.2.3 Turmeric

*Curcuma longa L.* is a valuable botanical resource, serves as a natural flavor and color additive, and prevents the pathological progression of chronic illnesses through its anti-oxidative and anti-inflammatory bioactivities. Curcumin, the active constituent of turmeric, is a lipophilic polyphenolic molecule. Research evidence indicates that its possible systemic health benefits may arise from its selective positive regulatory impact on gastrointestinal function (Qin et al., 2024). Curcumin stops lipid metabolic disorders from developing by modulating the gut microbiota to regulate the BAs-TGR5 signaling axis.

Curcumin supplementation reduces M1 (CD80, CD38, CD11c, *p* < 0.05) and M2 (Arginase-1, *p* = 0.0013) macrophage markers’ mRNA levels and downregulates adipose tissue inflammation, including p65 (*p* < 0.05), signal transducer and activator of transcription 1 (STAT1, *p* < 0.05), TLR4 (*p* < 0.05), and IL-6 (*p* < 0.05), in WAT. These metabolic effects in obese mice are mediated by changes in the proportionate quantity of the *Lactococcus*, *Parasutterella*, and *Turicibacter* (*p* < 0.05) and by converting curcumin into curcumin-O-glucuronide (Islam et al., 2021). The same conclusion is that curcumin promotes the growth of *Akkermansia*, *Bacteroides*, *Parabacteroides*, *Alistipes*, *Alloprevotella*, and *Clostridium clusters IV and XIVa* (*p* < 0.05) and controls the development of endotoxin-producing *Desulfovibrio* bacteria (*p* < 0.05) in obese mice (Li et al., 2021; Bertoncini-Silva et al., 2023). This microbiota rebuilding effectively ameliorates hepatic steatosis, inflammation of adipose tissue, and IR by regulating metabolic products.

A correlational study shows that the microbiota in the gut was associated with the enhanced effect of curcumin in raising circulating levels of DCA (*p* < 0.05) and LCA (*p* < 0.05), which are effective endogenous ligands of G-protein coupled receptor 19 (TGR5). Meanwhile, it can enhance Ucp1-stimulated (*p* < 0.05) thermogenesis and initiate the thermogenic adipose tissue’s cAMP/PKA signaling cascade to treat obesity. Using gut microbiota depletion and fecal microbiota transplantation (FMT) experiments, causal evidence has confirmed that this mechanism is entirely dependent on the gut microbiota and requires TGR5 (effects abated in TGR5-deleted mice) (Han et al., 2021). Separately, another mechanistic study has shown that curcumin can serve as a naturally occurring FXR antagonist and TGR5 agonist to reduce *Lactobacillus* (*p* < 0.05) enrichment and mitigate metabolic issues associated with DCA buildup. It also undermines the advantage of the *Proteobacteria* and *Clostridiaceae*, normalizes the synthetic route of BAs, and increases L-cell proliferation and GLP-1 release (*p* < 0.05) (Tian et al., 2023).

When applying curcumin in NASH patients, decreased hepatic fat content (*p* < 0.001) was observed in patients after 24 weeks of curcumin treatment. This has a close relationship with the control of the TGR5 signaling pathway and BAs metabolism, both of which are dependent on gut microbiota. Specifically, curcumin increased serum DCA (*p* = 0.018), peripheral blood monocyte TGR5 (*p* < 0.05), and serum GLP-1 (*p* = 0.012) and substantially decreased the *Firmicutes/Bacteroidetes* ratio (*p* < 0.01) while enriching *Bacteroides* (*p* < 0.001) abundance (He et al., 2024).

### 4.3 Saponins, terpenoids, and alkaloids

Saponins, terpenoids, and alkaloids, three major classes of naturally occurring bioactive compounds, exhibit distinct mechanisms in modulating gut microbiota: alkaloids regulate intestinal pH and enzyme activity to favor beneficial bacteria over pathogenic ones; terpenoids affect pathogen energy metabolism; and saponins alter bacterial membrane permeability. These actions promote immune homeostasis, intestinal barrier function, and systemic health (Table 3).

**TABLE 3:** The role of saponins, terpenoids, and alkaloids targeting gut microbiota in lipid metabolic disorders.

	Ingredients	Source	Disease	Experimental model	Dose and duration	Study types and sequencing method	Mechanism	Microbiota findings	References
Saponins	Polygonatum sibiricum saponin	Polygonatum	T2DM	HFD and STZ-induced male ICR mice	1, 1.5, 2 g/kg/day; 8 weeks	*In vivo*, 16S rRNA sequencing analysis, metabolomics analysis	↓FBG ↑HDL-C,↓TC, TG, LDL-C ↑chlorogenic	↑*Lactobacillus, Lachnospiraceae_NK4A136_group, Intestinimonas* ↓the ratio of *Firmicutes/Bacteroidetes*	(Chai et al., 2021)
	Polygonatum sibiricum saponin	Polygonatum	T2DM	*In vivo*: HSHFD and STZ-induced male ICR mice; *In vitro*: HepG2 cell	*In vivo*: 1, 1.5, 2 g/kg/day; 8 weeks *In vitro*: 10, 50, 100, 500, 1,000 μg/mL	*In vivo*; *in vitro*	↓α-amylase, α-glucosidase ↑hexokinase, pyruvate kinase	↑*Lactobacillus, Bifidobacterium* ↓*Enterococcus, Enterobacteriaceae*	(Luo et al., 2020)
	Total Astragalus saponins	Astragalus	T2DM	HSHFD and STZ-induced male SD rats	80 mg/kg/day; 12 weeks	*In vivo*	↓FBG ↑HDL-C,↓TC, TG, LDL-C, GSP ↑insulin, C-peptide,↓HOMA-IR ↓IL-1β, TNF-α ↑IRS-1, PI3K, PDK1, AKT, Gys2 ↓GSK-3β Via PI3K/AKT/GSK-3β signaling pathway	↑*Bifidobacterium, Ruminococcaceae UCG-014* ↓*Lactobacillus, Turicibacter* ↓the ratio of *Firmicutes/Bacteroidetes*	(Ma et al., 2023)
	Ginsenoside Rb1	Panax quinquefolius Linn.	T2DM	C57BL/6J mice and HFD-induced Kkay mice	200 mg/kg/day; 6 weeks	*In vivo*, 16S rRNA sequencing analysis, metabolomics analysis	↓FBG ↓TC, TG ↓serum insulin, HOMA-IR ↓FFA	↑ *Parasutterella* ↓ *Odoribacter, Anaeroplasma*	(Zhou X. et al., 2023)
Terpenoids	Pachymic acid	Poria cocos	NAFLD	HFD-induced C57BL/6J mice	20, 40 mg/kg/day; 12 weeks	*In vivo*, 16S rRNA sequencing analysis, liver metabolomics analysis	↓TC, TG ↓IL-1β,IL-6,TNF-α ↓FASN, SREBP1c, SCD1 ↑PPARα, CPT1α ↑oleic Via LPS/TLR4/MYD88/NFκB signaling pathway	↑*Akkermansia* ↓*Desulfovibrio, Streptococcus* ↓the ratio of *Firmicutes/Bacteroidetes*	(Ren et al., 2025)
	Mogroside-rich extract	Siraitia grosvenorii	Obesity	HFD-induced male C57BL/6J mice	300, 600 mg/kg/day; 18 weeks	*In vivo*, 16S rRNA sequencing analysis	↓Body weight, iWAT ↑insulin sensitivity	↓*Ruminiclostridium, Oscillibacter* ↓the ratio of *Firmicutes/Bacteroidetes*	(Wang et al., 2022c)
		Mogrosides (SG and L-SGgly)	Siraitia grosvenorii	T2DM	HFD and STZ-induced male SD rats	SG: 500 mg/kg/day L-SGgly: 20 mg/kg/day; 14 days	*In vivo*, 16S rRNA sequencing analysis, metabolite profiling analysis	↑SCFAs (acetic acid, propionic Acid, butyric acid) ↓deoxycholic acid, 1β-hydroxycholic acid	↑*Elusimicrobium, Acetitomaculum* ↓*Lachnospiraceae_UCG-004, Family_ XIII_ AD3011_ Group, Bilophila*	(Zhang et al., 2021a)
	Ganoderic acid A	Ganoderma lucidum	Hyperlipidemia	HFD-induced male Kunming mice	15, 75 mg/kg/day; 8 weeks	*In vivo*, 16S rRNA sequencing analysis, metabolomics analysis	↓body weight ↓TC, TG, LDL-C, NEFA, BA ↑SCFAs (acetic acid, propionic acid) ↓HMGCR, SREBP-1c, FAS, ACC1, ACAT2, C/EBPα, CD36, FATP ↑PPARα, FXR, Cyp7a1	↑*Eisenbergiella, Alistipes, Oscillibacter, Marvinbryantia, Bacteroides, Mucispirillum* ↓*Parabacteroides, Anaerotruncus, Barnesiella, Lactobacillus, Clostridium*	(Guo et al., 2020)
Alkaloids	1-Deoxynojirimycin (DNJ)	Mulberry leaf	Hyperlipidemia	*In vivo*: HFD and STZ-induced male and female ICR mice; *In vitro*: HepG2 cell	MLE:200, 400 mg/kg/day; DNJ:50 mg/kg/day; IPA:100 mg/kg/day; 12 weeks	*In vivo*, 16S rRNA sequencing analysis, metabolomics analysis; *In vitro*	↓Body weight ↓Glu, TC, TG, LDL-C ↓SREBP1c, FAS, ACC, SREBP2, HMGR ↑IPA	↑*Akkermansia, Clostridium XIVa*	(Li R. et al., 2020)
	Nuciferine	Lotus leaf	Obesity	HFD-induced male SD rats	10 mg/kg/day; 8 weeks	*In vivo*, 16S rRNA sequencing analysis, metabolomics analysis	↓Body weight, fat accumulation ↓TC, TG, LDL-C, ALT, AST ↓TNF-α, IL-1β, IL-6,↑IL-10 ↓FAS, SREBP-1, PPARγ ↑SCFAs (acetic acid, butyric acid) ↑TJ (ZO-1, Occludin) ↓LPS	↓*Desulfovibrio, Lachnospiraceae_* *NK4A136_group, Christensenellaceae_R-7_group, Allobaculum* ↓the ratio of *Firmicutes/Bacteroidetes*	(Wang Y. et al., 2020)

#### 4.3.1 Saponins: polygonatum

Polygonatum is the dry rhizome of plant species of the family Liliaceae, such as *Polygonatum cyrtonema Hua., Polygonatum sibiricum Red.*, or *Polygonatum kingianum Coll. et Hemsl*. To develop the main benefits of tonifying the qi, feeding the yin, strengthening the spleen, and moistening the lungs, Polygonatum undergoes a special processing method known as the nine steam-nine sun-dries, which is used to reduce the irritating ingredients in raw products and promote the conversion of active substances. Steroidal saponins are abundant in the rhizomes of Polygonatum plants, mainly diosgenin, yamogenin, and their analogs as aglycones, with sugar chains composed of one to four molecules of galactose, glucose, xylose, rhamnose, or fucose (Shi et al., 2023). Polygonatum saponins have a particular advantage in improving gut microbiota health and maintaining serum lipoprotein levels, as they influence the gut microbiota-lipid metabolism axis (Liu R. et al., 2024).

PSS increases IR-HepG2 cells’ intake of glucose and improves intracellular glycogen levels and the activities of hexokinase and pyruvate kinase (*p* < 0.05). In addition, PSS could prevent α-amylase and α-glucosidase (*p* < 0.05) from functioning. PSS reduced *Enterobacteriaceae*, *Pseudomonas*, and *Clostridium perfringens* (*p* < 0.05) and enhanced *Bifidobacterium*, *Lactobacillus*, *Lachnospiraceae_NK4A136_group*, and *Intestinimonas* (*p* < 0.05) in T2DM mice. It suppresses inflammatory responses by reducing the proportion of anaerobic, facultative anaerobic, potentially pathogenic, and Gram-negative bacteria. PSS significantly affects gut microbial function at the metabolic level, particularly by regulating carbohydrate and amino acid metabolites (including L-alanine and L-glutamic acid, *p* < 0.01) and by significantly elevating metabolite concentrations, such as inositol and chlorogenic acid (*p* < 0.01). PSS, the positive regulation of the gut microbiome, decreases the levels of FBG and insulin secretion (*p* < 0.05) in T2DM mice. The levels of TC, TG, and LDL-C in the blood were decreased (*p* < 0.01). In contrast, the content of HDL-C (*p* < 0.01) was increased (Luo et al., 2020; Chai et al., 2021). Therefore, PSS improved the metabolism of glucose and lipids and beneficially reduced resistance to insulin.

#### 4.3.2 Terpenoids: luo han guo

The dried fruit of *Siraitia grosvenorii Swingle C. Jeffrey ex A. M. Lu et Z. Y. Zhang* is referred to as Luo Han Guo. It is renowned for the wealth of secondary metabolites with intrinsic antioxidant properties. Some of the bioactive substances that are highly enriched in its pulp include terpenoids and amino acids. Of these, mogrosides are naturally occurring triterpenoid compounds found in Luo han guo that have attracted considerable attention for their role in conferring the fruit its characteristic sweetness and associated health benefits. The action targets of mogrosides are associated with oxidative damage that predominantly involves pathways in cancer, atherosclerosis, and lipid metabolism (Liu H. et al., 2025). Mogrosides are highly effective in improving lipid metabolism disorders; this effect is strictly linked to the presence of a healthy intestinal microbiota.

Mogrosides intervention restores the *Firmicutes/Bacteroidetes* ratio (*p* < 0.001) and reduces the relative quantity of *Ruminococcus* and *Oscillibacter* in obese mice, significantly decreasing weight gain, adipose tissue deposition, and fatty liver lesions, while improving insulin sensitivity and glucose tolerance (Wang et al., 2022c). These benefits are likewise observed in rats with T2DM. Mogrosides enhanced the relative number of *Elusimicrobium* (*p* < 0.01) and *Acettomaculum* (*p* < 0.001) and diminished the relative number of *Lachnospiraceae_UCG-004*, *Bilophila*, *Proteobacteria* (*p* < 0.05), and *Family_XIII_AD3011_group* (*p* < 0.001). Mogrosides also strongly elevated levels of acetate, propionate, butyrate, valeric acid, and hexanoic acid in the stool (*p* < 0.001) and also reduced the content of DCA, 1β-hydroxycholic acids, 6β-hydroxymedroxyprogesterone, and glycohyocholic acid (*p* < 0.05). Eventually, this further influenced GLP-1 levels, thereby controlling blood glucose and IR (Zhang et al., 2021a).

#### 4.3.3 Alkaloids: lotus leaf

Lotus leaf is the dried leaf of the lotus plant (*Nelumbo nucifera Gaertn*), a member of the Nymphaeaceae family. The key active constituents that positively impact glucose and fat absorption and metabolism include polysaccharides, organic acids, terpenoids, alkaloids, flavonoids, and volatile oils. Nuciferine (NF), roemerine, and O-nornuciferine are the three known alkaloid monomers that were extracted from lotus leaves. NF is the most remarkable with its biological activity and chemical properties (Liu H. et al., 2025). NF is an aporphine alkaloid that has a variety of pharmacological characteristics, including anti-inflammatory, anti-tumor, anti-obesity, anti-dyslipidemia, anti-hyperglycemia, and anti-hypouricemia, and it exhibits affinity for neural receptors, thereby preventing metabolism-related disorders. Its potential pathways include effects on Ca^2+^ flux, gut microbiota, ferroptosis, and targets and signaling pathways involved in metabolism, inflammation, and cancer (Wan Y. et al., 2022). Lotus leaf alkaloids (NUC) can alter the functional profile of the gut microbiota, which in turn modulates host metabolic pathways involving glycan biosynthesis and metabolism, as well as the metabolism of fats and carbohydrates.

NUC decreased the number of *Desulfovibrio*, *Allobaculum, Lachnospiraceae_NK4A136_group*, and *Christensenellaceae_R-7_group* (*p* < 0.05) and increased the number of *Prevotella_9* and *Bacteroides* (*p* < 0.05). NUC reduced the levels of expression of FASN (*p* < 0.01), SREBP (*p* < 0.001), and PPARγ (*p* < 0.01) and increased the PPARα (*p* < 0.05) expression level. Meanwhile, the decreased differentially metabolites belonged to lysophospholipids (LysoPCs), phospholipids (PCs), phosphatidylethanolamines (PEs), and lysobisphosphatidic acids (LPAs). In addition, six metabolites, comprising LysoPC (16:0), LysoPC (P-18:1), PE (18:1/24:1), PE (22:1/P-18:1), linolenic acid, and phosphatidylglycerophosphate (PGP) (16:0/22:5), were significantly increased. NUC exerts anti-inflammatory and metabolic-improving effects by increasing the number of microbiota to produce acetic acid and butyric acid, reducing the number of microbiota to produce LPS, enhancing intestinal barrier integrity by elevating ZO-1 and occludin (*p* < 0.05) expression, and finally suppressing the expression and secretion of IL-6, IL-1β, and TNF-α (*p* < 0.05) (Wang Y. et al., 2020). The administration of NUC prevented weight gain, reduced fat buildup, and improved the metabolism of lipids in an obese rat.

### 4.4 Limitations of preclinical studies

By regulating microbial enzyme activity and metabolite levels, compounds derived from MFH act as multi-target modulators that affect host lipid metabolism. Polysaccharides such as those from Astragalus, Lycium, and Poria reshape the gut microbiota, thereby increasing SCFA production. These SCFAs then enhance intestinal barrier integrity by activating GPR41/43 and upregulating tight junction proteins, including ZO-1. Polysaccharides have high molecular weights and complex structures, limiting their bioavailability in the upper gut. As a result, they must be fermented by colonic microbiota (Chen et al., 2024; Gao et al., 2024). Moreover, current studies vary widely in extraction methods, purity, dosing, and treatment length, making comparisons difficult. For example, APS have been administered at dietary supplementation levels of 2–8% or doses of 200–600 mg/kg, yet standardization of key physicochemical properties—such as molecular weight distribution, monosaccharide composition, and glycosidic linkage patterns—is lacking. This variability hinders cross-study comparisons and the establishment of reliable structure-activity relationships.

Polyphenolic compounds, such as curcumin, can act as FXR antagonists and TGR5 agonists, thereby linking gut microbiota changes to host thermogenesis and bile acid homeostasis. However, most polyphenols are rapidly metabolized and poorly absorbed, and their bioactivity often depends on extensive gut microbiota-mediated biotransformation to generate bioactive small-molecule metabolites (Ritika et al., 2024; Tosi et al., 2025). Consequently, the clinical efficacy of polyphenols is likely influenced by the host’s individual microbial metabolic capacity. In contrast to the research on polysaccharides and polyphenols in microbiota–host metabolic interactions, research on saponins, alkaloids, and terpenoids remains comparatively fragmented, lacking systematic preclinical trials and mechanistic validation.

Most studies report microbial compositional shifts only at the phylum or genus level without identifying specific bacterial strains or functional genes responsible for fermenting natural compounds and generating metabolites. Therefore, in many studies, the causal relationship between microbial alterations and metabolic benefits can only be inferred from correlations, rather than being rigorously validated using host-specific gene knockouts (e.g., GPR41/43, TGR5, FXR), FMT, or germ-free animal models. Furthermore, systematic investigations into bidirectional host–microbe co-metabolism remain scarce—particularly regarding whether gut microbial glycosidases hydrolyze natural compounds into bioactive oligosaccharides or aglycones and whether these intermediates directly regulate host signaling pathways independently of microbial activity. The spatiotemporal dynamics of microbiota remodeling across different gut regions (e.g., ileum vs. colon) are largely unexplored. Moreover, potential dose-dependent adverse effects and the possibility that certain natural compounds may enrich pathogenic or metabolically toxic commensals have not been systematically evaluated.

### 4.5 Clinical research

**FIGURE 5:**
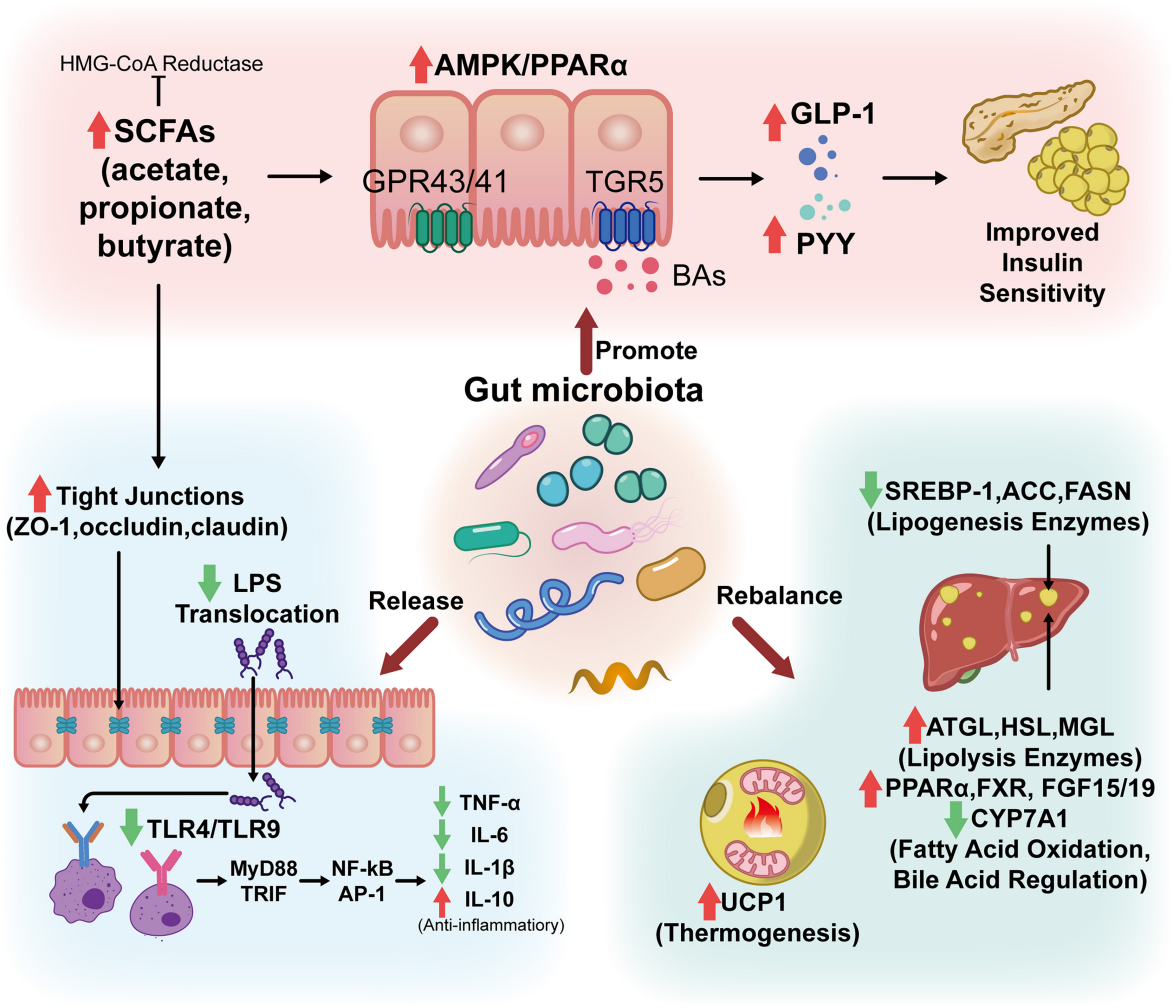
Regulation of the metabolism of lipids by natural compounds targeting gut microbiota in MFH substances. Natural compounds enhance the production of butyrate, acetate, and propionate by modifying the gut flora. Acetate and propionate cause intestinal L cells to release PYY and GLP-1 through GPR41/GPR43, which controls blood sugar and appetite. Acetate is a substrate for fatty acid synthesis and cholesterol, whereas propionate inhibits cholesterol synthesis by suppressing the activity of HMG-CoA reductase, thereby maintaining endogenous cholesterol homeostasis. Butyrate helps to preserve intestinal barrier integrity and decrease LPS translocation by raising ZO-1, occludin, and claudin expression. LPS binds to CD14/TLR4 on immune cell surfaces after entering the blood circulation. It promotes the production of pro-inflammatory cytokines, among them IL-6, IL-1β, and TNF-α by activating transcription factors like NF-κB and AP-1 through MyD88/TRIF-dependent pathways. Natural compounds influence bile acid metabolism by modulating the microbiota. FGF15/19 secretion is triggered by intestinal bile acids, activating the FXR. This inhibits hepatic CYP7A1 transcription and decreases bile acid production. Simultaneously, they lower the synthesis of TG and FAs by suppressing the production of SREBP1c and its downstream substrates, ACC and FASN. Additionally, they promote fatty acid oxidation by upregulating lipolysis-related enzymes and PPARα. Through the TGR5-cAMP-PKA signaling pathway, bile acids also cause intestinal L cells to release GLP-1, which regulates the secretion of insulin and glucagon. Moreover, natural compounds enhance fat consumption and energy metabolism by encouraging adipocytes to express UCP1 through microbiota-host interactions.

Recent clinical trials have shown that the presence of natural compounds in MFH substances can help improve abnormalities of lipid metabolism by regulating the intestinal microbiota, which serves as an intermediary (Figure 5). These are the active MFH substances that have shown evident probiotic activity in human clinical trials (Table 4).

**TABLE 4:** The role of MFH substance targeting gut microbiota in patients with lipid metabolism disorders.

Ingredients	Source	Disease	Experimental model	Dose and duration	Study types and sequencing method	Mechanism	Microbiota findings	References
Curcumin	Curcuma longa	NAFLD	Human patients	500 mg/day; 24 weeks	*In vivo*, 16S rRNA sequencing analysis, metabolomics analysis	↓CAP, body weight, BMI ↓FFA, FBG, TC, HbA1c, INS ↑TGR5, GLP-1	↑*Bacteroides* ↓the ratio of *Firmicutes/Bacteroidetes*	(He et al., 2024)
Alginate	Seaweed	Obesity	Human patients	15 g/day; 14 weeks	*In vivo*, 16S rRNA sequencing analysis	↓Body weight, BMI, hip circumference ↓ghrelin, neuropeptide Y ↑SCFAs ↑taurocholate acid, taurochenodeoxycholic acid Via primary and secondary bile acid biosynthesis signaling pathways	↓*Bacteroides, Oscillibacter, Eubacterium*	(Zhou X. et al., 2023)
Sulfated xylorhamnoglucuronan	Seaweed	Overweight	Human patients	2, 4 g/day; 12 weeks	*In vivo*	↓non-HDL-C, atherogenic index, 2-h insulin ↓CRP ↓IFN-γ, IL-1β, TNF-α, IL-10	↑*Bifidobacteria, Akkermansia, Pseudobutyrivibrio, Clostridium* ↓*Bilophila*	(Roach et al., 2022)
Almonds	Almond	Obesity	Human patients	56 g/day; 8 weeks	*In vivo*, 16S rRNA sequencing analysis	↓Fecal pH, moisture content	↑*Ruminococcaceae*	(Choo et al., 2021)
Sea buckthorn berry puree	Sea buckthorn berries	Hypercholesterolemia	Human patients	90 g/day; 90 days	*In vivo*, 16S rRNA sequencing analysis, plasma metabolomic analysis	↓ApoA-I, ApoB, MDA ↑Valine, ↓Glucose, lactate ↓Alanine ↓Creatine, Creatinine, Glutamine, Glutamate ↑Histidine,↓Serine	↑*Prevotella, Anaerostipes, Ruminococcus, Oscillibacter, Butyrivibrio, Blautia, Eubacterium, Faecalibacterium, Ruminococcaceae, Sporobacter* ↓*Parasutterella*	(Chen K. et al., 2022)
Red raspberry (RRB)	Red raspberry	Prediabetes	Human patients	RRB smoothie; 16 weeks	*In vivo*, Shotgun metagenomic sequencing analysis	↓Hepatic-IR, TC, LDL-C	↑*Eubacterium eligens* ↓*Ruminococcus gnavus*	(Zhang et al., 2022)

Patients who were supplemented with 500 mg of curcumin daily showed an increase in the relative number of *Bacteroides* within the gut and a drop in the *Firmicutes/Bacteroidetes* ratio after 24 weeks in 80 patients with NAFLD. DCA (*p* = 0.018) levels were also increased with curcumin treatment, as well as GLP-1 (*p* = 0.012) expression. TGR5/GLP-1 pathway was activated, and controlled attenuation parameters (CAPs, *p* < 0.001) and body mass index (BMI, *p* = 0.032) were significantly reduced. Further, TC (*p* = 0.001), FBG (*p* = 0.038), hemoglobin A1c (HbA1c, *p* = 0.019), and insulin (*p* = 0.043) were also significantly lower (He et al., 2024). Among an 80-participant overweight cohort randomized to receive 15 g of alginate daily, 14 weeks of intervention elevated fecal conjugated bile acids (taurocholic acid and taurochenodeoxycholic acid, *q* < 0.10) and decreased bile acid-associated bacteria (*Bacteroides*, *Oscillibacter*, and *Eubacterium*, *q* < 0.20). It also reduced body fat and BMI (*p* < 0.05), as well as appetite hormones (ghrelin and neuropeptide Y) (Zhou X. et al., 2023). Sixty-four overweight individuals (median age of 55 years, median BMI of 29 kg/m^2^) were supplemented daily with 2 g or 4 g of 84-derived sulfated polysaccharide-xylorhamnoggucuronan (SXRG84) for 6 weeks. The findings indicated substantial similarities in gut microbiota, with *Bifidobacteria*, *Akkermansia*, *Pseudobutyrivibrio*, and *Clostridium* increasing and *Bilophila* decreasing. Non-HDL cholesterol (*p* = 0.02), atherosclerosis warning signs (*p* = 0.05), and 2-h insulin levels (*p* = 0.02) showed a decline in participants given 2 g/day. C-reactive protein (CRP) declined substantially (*p* = 0.03) in participants who received 4 g/day. The levels of interferon-γ (IFN-γ), IL-1β, and TNF-α ameliorated significantly following a 12-week course of therapy (*p* < 0.05) (Roach et al., 2022).

Although natural compounds from MFH have shown considerable promise in preclinical models for treating lipid metabolism disorders, significant differences remain before these findings can be translated into clinical practice. In preclinical studies, natural bioactive substances are often used as crude extracts or traditional formulations. In contrast, human clinical trials typically employ highly purified or standardized formulations, such as purified curcumin (500 mg), standardized alginate (15 g), or sulfated extract SXRG84 (2–4 g). This shift from complex natural matrices to isolated compounds has created a disconnect between preclinical and clinical research. Moreover, purified compounds may interact with the gut microbiota differently than their natural counterparts, potentially altering their bioactivity and therapeutic effects.

In order to close the current gap, future research needs to focus on comparing the effects of standardized products versus crude extracts under human physiological conditions, despite some positive clinical results, such as purified curcumin increasing *Bacteroides* abundance and GLP-1 levels in NAFLD patients. The limitations of current clinical data make this problem even more difficult. The majority of trials have small sample sizes and short durations and fail to account for individual differences in the gut flora, which is a crucial factor impacting therapy response. Additionally, the absence of standardized dosing and administration protocols between preclinical and clinical studies hinders the translation of research into reliable clinical guidance.

Apart from the translational disconnect caused by formulation simplification and altered microbial interactions, the clinical transferability of the doses employed in animal studies is another key challenge. The levels at which therapeutic efficacy is observed in rodents for many natural compounds derived from MFH substances may not be readily achievable within the established safe range for human intake when converted to human-equivalent doses (HED) based on body surface area. For instance, the dosage for DOPs is 200 mg/kg/day (Liu et al., 2023), Lycium barbarum polysaccharides is 400 mg/kg/day (Lu et al., 2021), and Astragalus membranaceus polysaccharides is 600 mg/kg/day (Song et al., 2022). HED (mg/kg) = Animal effective dose (mg/kg) × (Animal Weight/Human Weight)^ 0.33. The aforementioned doses, assuming a mouse weighs 40 g, translate into daily human doses of around 1, 2, and 3 g/day for an adult weighing 60 kg. This discrepancy in dosage suggests that doses high enough to dramatically alter the gut microbiota and lipid metabolism in animal models might not be immediately applicable to human clinical settings. As a result, even though these animal studies offer important molecular insights, their clinical transferability is limited.

Multiple lines of evidence support the potential of MFH compounds to modulate lipid metabolism. Preclinical studies indicate that these compounds can reshape the gut microbiota and regulate host lipid pathways. Human randomized controlled trials involving compounds such as curcumin and alginates have further confirmed their ability to reduce body mass index, improve inflammatory markers, and alter the composition of the gut microbiota. However, clinical translation remains challenging: when animal-derived effective doses are converted to human-equivalent doses based on body surface area, they often fall outside the conventional safe intake range. This discrepancy—often an order of magnitude—combined with a lack of dose standardization, hinders the direct application of preclinical findings to clinical practice.

## 5 Conclusion and outlook

The natural compounds, particularly those sourced from the MFH substance, are gaining increasing recognition for their potential in disease prevention and health maintenance. These compounds are considered the most ideal choices for controlling lipid metabolism-related disorders such as obesity, diabetes, and NAFAD due to their broad pharmacological properties, low toxicity, high biocompatibility, and the diversity of bioactive compounds. This review highlights that the ability of natural compounds present in MFH substances to regulate the gut microbiome may underlie their therapeutic effects on lipid metabolism-related disorders. These naturally occurring agents have now been demonstrated to have therapeutic benefit in animal models, cell studies, and some clinical studies. By altering gut microbiota composition and subsequent microbial metabolite production, such substances can influence host fatty acid synthesis and catabolism, thereby potentially retarding the progression of metabolic diseases.

**FIGURE 6:**
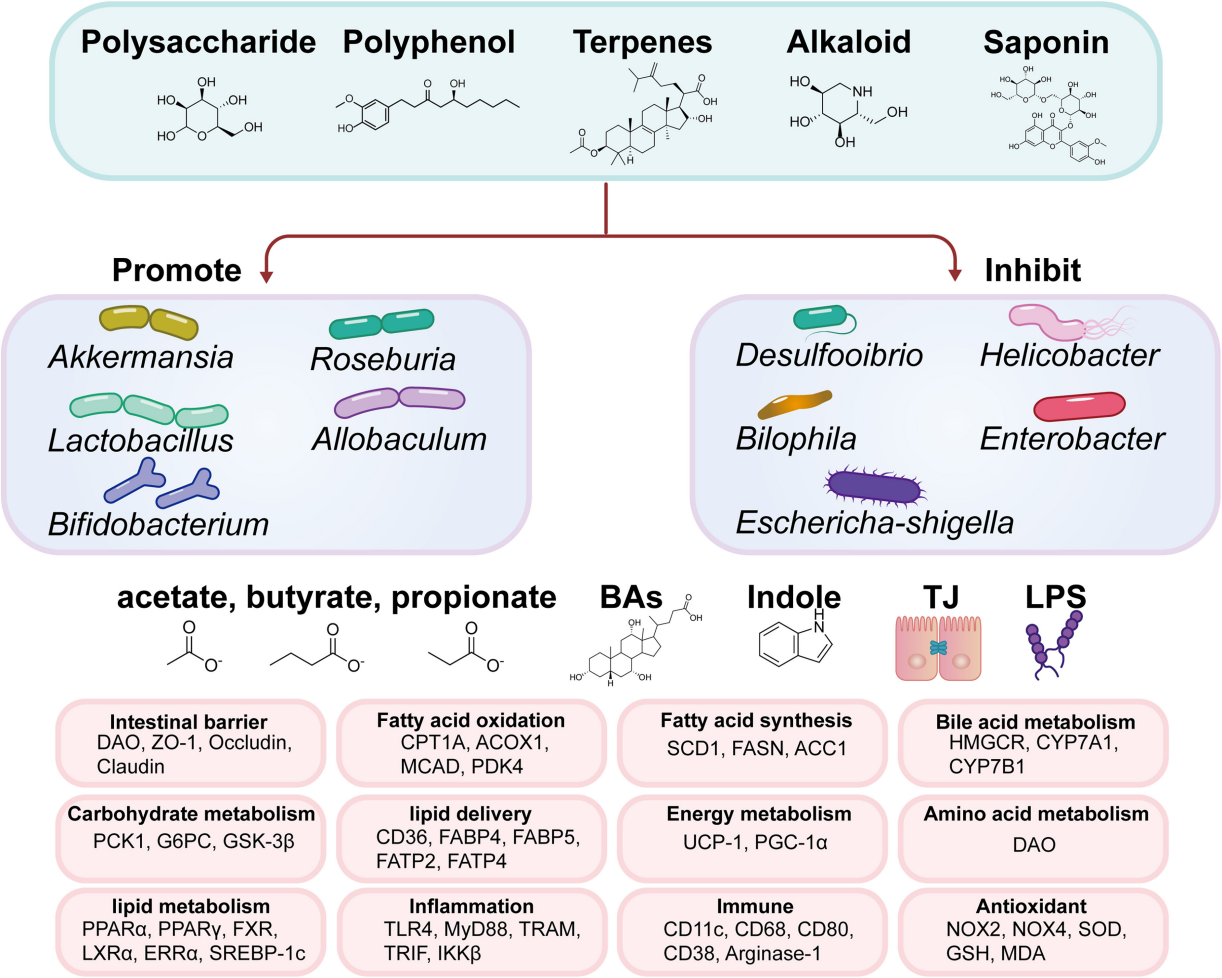
Potential mechanisms of natural compounds in MFH substances regulating lipid metabolism. By altering the microbiota’s composition and its metabolites (such as BAs, SCFAs, D-lactate, formic acid, IPA, etc.), natural compounds in MFH substances can affect gut barrier-related proteins (DAO, ZO-1, Occludin, Claudin); enzymes associated with fatty acid oxidation (CPT1A, ACOX1, MCAD, PDK4), fatty acid synthesis (SCD1, FASN, ACC1), cholesterol and bile acid metabolism (HMGCR, CYP7A1, CYP7B1), carbohydrate metabolism (PCK1, G6PC, GSK-3β); lipid delivery (CD36, FABP4, FABP5, FATP2, FATP4); energy metabolism regulators (UCP-1, PGC-1α); amino acid metabolism (DAO); lipid metabolism-related transcription factors (SREBP-1c, PPARα, PPARγ, FXR, LXRα, ERRα); inflammation-related molecules (TLR4, MyD88, TRAM, TRIF, IKKβ); immune-related molecules (CD11c, CD68, CD80, CD38, Arginase-1); oxidative stress and antioxidant-related molecules (NOX2, NOX4, SOD, GSH, MDA). Ultimately, this regulates lipid metabolism by adjusting intestinal barrier integrity, fatty acid oxidation and synthesis, cholesterol metabolism, lipogenesis, and inflammatory responses.

Natural compounds in MFH substances promote the growth of beneficial bacteria (e.g., *Akkermansia*, *Allobaculum*, and *Parabacteroides*) while reducing harmful ones (e.g., *Desulfovibrio*, *Enterococcus*, and *Escherichia-Shigella*). After microbiota restructuring, their metabolites include BAs, SCFAs, LPS, BCAAs, indoles, and their derivatives. By influencing intestinal barrier-related proteins, metabolic pathways linked to lipid delivery, energy metabolism regulators, and related enzymes such as fatty acid oxidation, fatty acid synthesis, cholesterol and bile acid metabolism, carbohydrate metabolism, and amino acid metabolism; transcription factors linked to lipid metabolism that are involved in metabolic regulation; and molecules related to inflammation, immunology, oxidative stress, and antioxidants that are connected to alterations in the intracellular and extracellular environments. In addition, MFH natural compounds improve energy metabolism, mitigate oxidative stress, mitigate metabolic endotoxemia, and regulate fatty acid and bile acid metabolism. Collectively, these multimodal mechanisms provide a theoretical foundation and novel nutritional intervention strategies to exert probiotic effects via diets based on natural compounds in MFH substances and develop therapeutic agents for metabolic diseases (Figure 6).

According to available data, natural compounds in MFH substances can beneficially influence metabolic diseases by adjusting gut microenvironment homeostasis, secreting bioactive metabolites, and participating in host–microbiota interaction, with generally no adverse side effects. Nevertheless, the complexity of interactions among natural compounds, the host, and the gut flora poses a challenge for clinical application.

Future studies ought to prioritize elucidating the four factors listed below to advance our understanding of how natural compounds in MFH substances modulate the gut microbiota and host physiology and to provide practical direction for therapeutic applications.

(a) We gathered 50 studies on how extracts from MFH substances ameliorate lipid metabolic diseases by balancing gut flora. These investigations primarily aim to observe the effects of the extracts as a whole. Research on the unique structural frameworks, diverse bioactivities, and mechanism-specific natural compounds within these extracts remains insufficient and warrants further investigation. Additionally, it is needed to identify natural compounds with specific strain-generating capabilities and the particular molecular targets, as well as host-dependent regulatory mechanisms from existing studies.
(b) Most current research relies on 16S rRNA sequencing to analyze therapy-induced taxonomic shifts in the gut microbiota. While this method effectively identifies changes at the genus level, metabolic benefits are often strain-specific, and the resolution of 16S rRNA sequencing is generally insufficient to distinguish particular species. For instance, many studies have reported that an increased abundance of the genus *Akkermansia* is associated with improved lipid metabolism (Xia et al., 2021; Zheng et al., 2022). However, it remains unclear whether this effect is attributable to the beneficial probiotic species *Akkermansia muciniphila* or to less-characterized species such as *A. glyciphila* or *A. biwaensis*. Indeed, one important aspect through which the gut microbiota affects host lipid metabolism is functional differences at the strain level.

One important aspect that the gut microbiota affects host lipid metabolism is through functional differences at the strain level. Certain strains or species of the same genus may have quite distinct, and occasionally even opposing, effects on the health of the host. Using the genus *Desulfovibrio* as an example, administration of natural compounds derived from MFH typically reduces its abundance in various models of lipid metabolism disorders. Such a reduction is associated with the restoration of host-microbe symbiosis (Wang Y. et al., 2020; Li et al., 2021). However, APS specifically enrich *Desulfovibrio vulgaris* and, via the acetic acid they produce as a metabolic signaling molecule, inhibit the expression of FASN and CD36, thereby suppressing *de novo* lipid synthesis and lipid uptake in the liver (Hong et al., 2021). This strain-level functional heterogeneity is revealed by the differential effects of strains within the same genus. It further suggests that the functional impacts of the gut microbiota are highly specific to the therapeutic context: certain strains within a genus may play a protective role in lipid metabolism, while others may contribute to the development of metabolic diseases. Therefore, the therapeutic potential of natural compounds derived from MFH lies not in the broad-spectrum promotion or inhibition of specific taxonomic groups, but rather in the selective regulation of particular metabolic pathways within these groups. To this end, adopt modern technologies, such as genomics, proteomics, and metabolomics, to dynamically dissect the spatiotemporal restructuring characteristics of lipid metabolism networks during beneficial bacterial colonization, with a focus on elucidating the precise interventions of individual bacterial strains and their metabolites on the host.

(c) While fecal samples are widely used, they provide only limited knowledge of the microbial communities in the terminal gut. Such samples are unable to adequately reflect the functioning and compositional dynamics of microbial components, including fungi and viruses, nor to reveal the spatial distribution of microorganisms within different intestinal regions (e.g., the ileocecal region). In addition, *in vivo* microbial-host interaction scenarios are complex, and *in vitro* models struggle to mimic them. Therefore, adopting *in situ* sampling technology, multi-omics integration analysis, organoid models, and clinical research should be combined.
(d) Currently, our understanding of the mechanisms of action of MFH compounds is primarily derived from preclinical studies, while an increasing body of clinical research is providing crucial translational evidence that compensates for the limitations of the former. Integrating these clinical findings with preclinical molecular discoveries further clarifies the therapeutic potential of MFH compounds as modulators of human lipid metabolism disorders. However, future research should focus on establishing a coherent translational pathway from preclinical to clinical studies, prioritizing standardized human-equivalent doses and long-term longitudinal evaluations. In clinical trials, systematic evaluations are needed to assess the differences between crude extracts and standardized products regarding their effects on gut microbiota and metabolic markers. Therefore, trial designs should be optimized. This includes estimating the sample size based on effect sizes obtained from previous studies while accounting for subgroup analyses and dropout rates to appropriately increase the sample size. Additionally, extending the treatment period (e.g., to 12 weeks or longer) and incorporating subgroup analyses based on microbiome stratification to clarify the true mechanisms of synergistic effects and host-microbiome interactions. Concurrently, use clinical trial samples for multi-omics integrated analyses to observe individualized response patterns of the microbiome. Future research should focus on determining the minimum effective dose in humans, conducting rigorous pharmacokinetic and safety evaluations, and conducting extensive, long-term randomized controlled trials to evaluate the therapeutic value of natural compounds from MFH in treating disorders of lipid metabolism by modulating the microenvironment of the microbiota.

## References

[B1] Ağagündüz D. Icer M. A. Yesildemir O. Koçak T. Kocyigit E. Capasso R. (2023). The roles of dietary lipids and lipidomics in gut-brain axis in type 2 diabetes mellitus. *J. Transl. Med.* 21:240. 10.1186/s12967-023-04088-5 37009872 PMC10068184

[B2] Akhtar M. Chen Y. Ma Z. Zhang X. Shi D. Khan J. A. et al. (2022). Gut microbiota-derived short chain fatty acids are potential mediators in gut inflammation. *Anim. Nutr.* 8 350–360. 10.1016/j.aninu.2021.11.005 35510031 PMC9040132

[B3] An S. M. Cho S. H. Yoon J. C. (2023). Adipose tissue and metabolic health. *Diabetes Metab. J.* 47 595–611. 10.4093/dmj.2023.0011 37482656 PMC10555533

[B4] Asnicar F. Berry S. E. Valdes A. M. Nguyen L. H. Piccinno G. Drew D. A. et al. (2021). Microbiome connections with host metabolism and habitual diet from 1,098 deeply phenotyped individuals. *Nat. Med.* 27 321–332. 10.1038/s41591-020-01183-8 33432175 PMC8353542

[B5] Baars D. P. Fondevila M. F. Meijnikman A. S. Nieuwdorp M. (2024). The central role of the gut microbiota in the pathophysiology and management of type 2 diabetes. *Cell Host Microbe* 32 1280–1300. 10.1016/j.chom.2024.07.017 39146799

[B6] Bell L. Whyte A. Duysburgh C. Marzorati M. Van den Abbeele P. Le Cozannet R. et al. (2022). A randomized, placebo-controlled trial investigating the acute and chronic benefits of American Ginseng (Cereboost§) on mood and cognition in healthy young adults, including in vitro investigation of gut microbiota changes as a possible mechanism of action. *Eur. J. Nutr.* 61 413–428. 10.1007/s00394-021-02654-5 34396468 PMC8783888

[B7] Bertoncini-Silva C. Fassini P. G. Carlos D. de Paula N. A. Ramalho L. N. Z. Rodrigues Giuliani M. et al. (2023). The dose-dependent effect of curcumin supplementation on inflammatory response and gut microbiota profile in high-fat fed C57BL/6 Mice. *Mol. Nutr. Food Res.* 67:e2300378. 10.1002/mnfr.202300378 37818762

[B8] Brown E. M. Clardy J. Xavier R. J. (2023). Gut microbiome lipid metabolism and its impact on host physiology. *Cell Host Microbe* 31 173–186. 10.1016/j.chom.2023.01.009 36758518 PMC10124142

[B9] Cai J. Rimal B. Jiang C. Chiang J. Y. L. Patterson A. D. (2022). Bile acid metabolism and signaling, the microbiota, and metabolic disease. *Pharmacol. Ther.* 237:108238. 10.1016/j.pharmthera.2022.108238 35792223

[B10] Canfora E. E. Meex R. C. R. Venema K. Blaak E. E. (2019). Gut microbial metabolites in obesity, NAFLD and T2DM. *Nat. Rev. Endocrinol.* 15 261–273. 10.1038/s41574-019-0156-z 30670819

[B11] Cao C. Wang Z. Gong G. Huang W. Huang L. Song S. et al. (2022). Effects of *Lycium barbarum* polysaccharides on immunity and metabolic syndrome associated with the modulation of gut microbiota: a review. *Foods* 11:3177. 10.3390/foods11203177 37430929 PMC9602392

[B12] Chai Y. Luo J. Bao Y. (2021). Effects of *Polygonatum sibiricum* saponin on hyperglycemia, gut microbiota composition and metabolic profiles in type 2 diabetes mice. *Biomed. Pharmacother.* 143:112155. 10.1016/j.biopha.2021.112155 34517283

[B13] Chen K. Zhou F. Zhang J. Li P. Zhang Y. Yang B. (2022). Dietary supplementation with sea buckthorn berry puree alters plasma metabolomic profile and gut microbiota composition in hypercholesterolemia population. *Foods* 11:2481. 10.3390/foods11162481 36010480 PMC9407212

[B14] Chen W. Dong M. Wang L. Wu J. Cong M. Yang R. et al. (2024). In vitro digestive and fermentation characterization of *Polygonatum cyrtonema* polysaccharide and its effects on human gut microbiota. *LWT* 203:116346. 10.1016/j.lwt.2024.116346

[B15] Chen X. Chen C. Fu X. (2022). Hypoglycemic effect of the polysaccharides from *Astragalus membranaceus* on type 2 diabetic mice based on the “gut microbiota-mucosal barrier”. *Food Funct.* 13 10121–10133. 10.1039/d2fo02300h 36106494

[B16] Chen X. Chen C. Fu X. (2023a). Dendrobium officinale polysaccharide alleviates type 2 diabetes mellitus by restoring gut microbiota and repairing intestinal barrier via the LPS/TLR4/TRIF/NF-kB Axis. *J. Agric. Food Chem.* 71 11929–11940. 10.1021/acs.jafc.3c02429 37526282

[B17] Chen X. Tong Y. L. Ren Z. M. Chen S. S. Mei X. Y. Zhou Q. Y. et al. (2023b). Hypoglycemic mechanisms of *Polygonatum sibiricum* polysaccharide in db/db mice via regulation of glycolysis/gluconeogenesis pathway and alteration of gut microbiota. *Heliyon* 9:e15484. 10.1016/j.heliyon.2023.e15484 37128343 PMC10147986

[B18] Cheng H. Zhang D. Wu J. Liu J. Zhou Y. Tan Y. et al. (2023). Interactions between gut microbiota and polyphenols: a mechanistic and metabolomic review. *Phytomedicine* 119:154979. 10.1016/j.phymed.2023.154979 37552899

[B19] Choo J. M. Tran C. D. Luscombe-Marsh N. D. Stonehouse W. Bowen J. Johnson N. et al. (2021). Almond consumption affects fecal microbiota composition, stool pH, and stool moisture in overweight and obese adults with elevated fasting blood glucose: a randomized controlled trial. *Nutr. Res.* 85 47–59. 10.1016/j.nutres.2020.11.005 33444970

[B20] Creedon A. Dimidi E. Hung E. Scott M. Probert C. Berry S. et al. (2022). Almonds and their impact on gastrointestinal physiology, luminal microbiology and gastrointestinal function: a randomized controlled trial. *Curr. Dev. Nutr.* 6:1002. 10.1093/cdn/nzac069.007 PMC9761756 36130222

[B21] Creedon A. C. Dimidi E. Hung E. S. Rossi M. Probert C. Grassby T. et al. (2022). The impact of almonds and almond processing on gastrointestinal physiology, luminal microbiology, and gastrointestinal symptoms: a randomized controlled trial and mastication study. *Am. J. Clin. Nutr.* 116 1790–1804. 10.1093/ajcn/nqac265 36130222 PMC9761756

[B22] Crichton M. Marshall S. Marx W. Isenring E. Vázquez-Campos X. Dawson S. L. et al. (2023). Effect of ginger root powder on gastrointestinal bacteria composition, gastrointestinal symptoms, mental health, fatigue, and quality of life: a double-blind placebo-controlled trial. *J. Nutr.* 153 3193–3206. 10.1016/j.tjnut.2023.09.002 37690779

[B23] Cui R. Zhang C. Pan Z. H. Hu T. G. Wu H. (2024). Probiotic-fermented edible herbs as functional foods: a review of current status, challenges, and strategies. *Compr. Rev. Food Sci. Food Saf.* 23:e13305. 10.1111/1541-4337.13305 38379388

[B24] Dai H. Shan Z. Shi L. Duan Y. An Y. He C. et al. (2024). Mulberry leaf polysaccharides ameliorate glucose and lipid metabolism disorders via the gut microbiota-bile acids metabolic pathway. *Int. J. Biol. Macromol.* 282(Pt 2):136876. 10.1016/j.ijbiomac.2024.136876 39490871

[B25] Domingo-Fernández D. Gadiya Y. Preto A. J. Krettler C. A. Mubeen S. Allen A. et al. (2024). Natural products have increased rates of clinical trial success throughout the drug development process. *J. Nat. Prod.* 87 1844–1851. 10.1021/acs.jnatprod.4c00581 38970498 PMC11287737

[B26] Duan Y. Huang J. Sun M. Jiang Y. Wang S. Wang L. et al. (2023). Poria cocos polysaccharide improves intestinal barrier function and maintains intestinal homeostasis in mice. *Int. J. Biol. Macromol.* 249:125953. 10.1016/j.ijbiomac.2023.125953 37517750

[B27] Dyall S. C. Balas L. Bazan N. G. Brenna J. T. Chiang N. da Costa Souza F. et al. (2022). Polyunsaturated fatty acids and fatty acid-derived lipid mediators: recent advances in the understanding of their biosynthesis, structures, and functions. *Prog. Lipid Res.* 86:101165. 10.1016/j.plipres.2022.101165 35508275 PMC9346631

[B28] Esler W. P. Cohen D. E. (2024). Pharmacologic inhibition of lipogenesis for the treatment of NAFLD. *J. Hepatol.* 80 362–377. 10.1016/j.jhep.2023.10.042 37977245 PMC10842769

[B29] Falduto M. Smedile F. Zhang M. Zheng T. Zhu J. Huang Q. et al. (2022). Anti-obesity effects of Chenpi: an artificial gastrointestinal system study. *Microb Biotechnol.* 15 874–885. 10.1111/1751-7915.14005 35170866 PMC8913872

[B30] Fotschki B. Cholewińska E. Ognik K. Sójka M. Milala J. Fotschki J. et al. (2023). Dose-Related regulatory effect of raspberry polyphenolic extract on cecal microbiota activity, lipid metabolism and inflammation in rats fed a diet rich in saturated fats. *Nutrients* 15:354. 10.3390/nu15020354 36678224 PMC9865883

[B31] Fu Q. Huang H. Ding A. Yu Z. Huang Y. Fu G. et al. (2022). *Portulaca oleracea* polysaccharides reduce serum lipid levels in aging rats by modulating intestinal microbiota and metabolites. *Front. Nutr.* 9:965653. 10.3389/fnut.2022.965653 35983485 PMC9378863

[B32] Gangzheng W. Xianglian C. Chengyuan S. Huang Q. Zhang C. Lin M. et al. (2023). Gut microbiota and metabolite insights into anti-obesity effect of carboxymethyl pachymaran in high-fat diet mice. *J. Funct. Foods* 111:105898. 10.1016/j.jff.2023.105898

[B33] Gao L. L. Ma J. M. Fan Y. N. Zhang Y. N. Ge R. Tao X. J. et al. (2021). *Lycium barbarum* polysaccharide combined with aerobic exercise ameliorated nonalcoholic fatty liver disease through restoring gut microbiota, intestinal barrier and inhibiting hepatic inflammation. *Int. J. Biol. Macromol.* 183 1379–1392. 10.1016/j.ijbiomac.2021.05.066 33992651

[B34] Gao Y. Wang J. Xiao Y. Yu L. Tang Q. Wang Y. et al. (2024). Structure characterization of an agavin-type fructan isolated from *Polygonatum cyrtonema* and its effect on the modulation of the gut microbiota in vitro. *Carbohydrate Polym.* 330:121829. 10.1016/j.carbpol.2024.121829 38368108

[B35] Gu W. Wang Y. Zeng L. Dong J. Bi Q. Yang X. et al. (2020). Polysaccharides from *Polygonatum kingianum* improve glucose and lipid metabolism in rats fed a high fat diet. *Biomed. Pharmacother.* 125:109910. 10.1016/j.biopha.2020.109910 32028238

[B36] Guo R. Liao J. Sun Y. Wang Y. Li P. Du B. (2025). A review of advances in the extraction, structural characterization, gel properties, and biological activity mechanisms of *Dendrobium officinale* polysaccharides. *Int. J. Biol. Macromol.* 311(Pt 2):143756. 10.1016/j.ijbiomac.2025.143756 40318733

[B37] Guo W. L. Guo J. B. Liu B. Y. Lu J. Q. Chen M. Liu B. et al. (2020). Ganoderic acid A from *Ganoderma lucidum* ameliorates lipid metabolism and alters gut microbiota composition in hyperlipidemic mice fed a high-fat diet. *Food Funct.* 11 6818–6833. 10.1039/d0fo00436g 32686808

[B38] Han X. Zhao W. Zhou Q. Chen H. Yuan J. Xiaofu Z. et al. (2022). Procyanidins from hawthorn (*Crataegus pinnatifida*) alleviate lipid metabolism disorder via inhibiting insulin resistance and oxidative stress, normalizing the gut microbiota structure and intestinal barrier, and further suppressing hepatic inflammation and lipid accumulation. *Food Funct.* 13 7901–7917. 10.1039/d2fo00836j 35781311

[B39] Han Z. Yao L. Zhong Y. Xiao Y. Gao J. Zheng Z. et al. (2021). Gut microbiota mediates the effects of curcumin on enhancing Ucp1-dependent thermogenesis and improving high-fat diet-induced obesity. *Food Funct.* 12 6558–6575. 10.1039/d1fo00671a 34096956

[B40] He Y. Chen X. Li Y. Liang Y. Hong T. Yang J. et al. (2024). Curcumin supplementation alleviates hepatic fat content associated with modulation of gut microbiota-dependent bile acid metabolism in patients with nonalcoholic simple fatty liver disease: a randomized controlled trial. *Am. J. Clin. Nutr.* 120 66–79. 10.1016/j.ajcnut.2024.05.017 38795741

[B41] Hong T. Jiang X. Zou J. Yang J. Zhang H. Mai H. et al. (2022). Hepatoprotective effect of curcumin against bisphenol A-induced hepatic steatosis via modulating gut microbiota dysbiosis and related gut-liver axis activation in CD-1 mice. *J. Nutr. Biochem.* 109:109103. 10.1016/j.jnutbio.2022.109103 35780999

[B42] Hong Y. Li B. Zheng N. Wu G. Ma J. Tao X. et al. (2020). Integrated metagenomic and metabolomic analyses of the effect of astragalus polysaccharides on alleviating high-fat diet-induced metabolic disorders. *Front. Pharmacol.* 11:833. 10.3389/fphar.2020.00833 32587515 PMC7299173

[B43] Hong Y. Sheng L. Zhong J. Tao X. Zhu W. Ma J. et al. (2021). *Desulfovibrio vulgaris*, a potent acetic acid-producing bacterium, attenuates nonalcoholic fatty liver disease in mice. *Gut Microbes* 13 1–20. 10.1080/19490976.2021.1930874 34125646 PMC8205104

[B44] Horasan Sagbasan B. Williams C. M. Bell L. Barfoot K. L. Poveda C. Walton G. E. (2024). Inulin and freeze-dried blueberry intervention lead to changes in the microbiota and metabolites within in vitro studies and in cognitive function within a small pilot trial on healthy children. *Microorganisms* 12:1501. 10.3390/microorganisms12071501 39065269 PMC11279127

[B45] Huang J. Guan B. Lin L. Wang Y. (2021). Improvement of intestinal barrier function, gut microbiota, and metabolic endotoxemia in type 2 diabetes rats by curcumin. *Bioengineered* 12 11947–11958. 10.1080/21655979.2021.2009322 34818970 PMC8810160

[B46] Huang X. Zhou Y. Sun Y. Wang Q. (2022). Intestinal fatty acid binding protein: a rising therapeutic target in lipid metabolism. *Prog Lipid Res.* 87:101178. 10.1016/j.plipres.2022.101178 35780915

[B47] Huang Y. Hu J. Xia Q. Tang M. Wang Y. Wang G. et al. (2024). Amelioration of obesity and inflammation by polysaccharide from unripe fruits of raspberry via gut microbiota regulation. *Int. J. Biol. Macromol.* 261(Pt 2):129825. 10.1016/j.ijbiomac.2024.129825 38309402

[B48] Islam T. Koboziev I. Albracht-Schulte K. Mistretta B. Scoggin S. Yosofvand M. et al. (2021). Curcumin reduces adipose tissue inflammation and alters gut microbiota in diet-induced obese male mice. *Mol. Nutr. Food Res.* 65:e2100274. 10.1002/mnfr.202100274 34510720

[B49] Jardon K. M. Canfora E. E. Goossens G. H. Blaak E. E. (2022). Dietary macronutrients and the gut microbiome: a precision nutrition approach to improve cardiometabolic health. *Gut* 71 1214–1226. 10.1136/gutjnl-2020-323715 35135841 PMC9120404

[B50] Jiang S. Liang C. Wan X. Po Lai K. Li R. Chen J. et al. (2024). Integrative analysis reveals the anti-obesity roles of *Poria cocos* polysaccharides through beneficial effects on gut microbiota. *J. Funct. Foods.* 119:106308. 10.1016/j.jff.2024.106308

[B51] Jiang W. Tan J. Zhang J. Deng X. He X. Zhang J. et al. (2024). Polysaccharides from *Dendrobium officinale* improve obesity-induced insulin resistance through the gut microbiota and the SOCS3-mediated insulin receptor substrate-1 signaling pathway. *J. Sci. Food Agric.* 104 3437–3447. 10.1002/jsfa.13229 38111200

[B52] Jinnouchi M. Miyahara T. Suzuki Y. (2021). Coix seed consumption affects the gut microbiota and the peripheral lymphocyte subset profiles of healthy male adults. *Nutrients* 13:4079. 10.3390/nu13114079 34836336 PMC8618347

[B53] Kane J. P. Pullinger C. R. Goldfine I. D. Malloy M. J. (2021). Dyslipidemia and diabetes mellitus: role of lipoprotein species and interrelated pathways of lipid metabolism in diabetes mellitus. *Curr. Opin. Pharmacol.* 61 21–27. 10.1016/j.coph.2021.08.013 34562838

[B54] Ke W. Flay K. J. Huang X. Hu X. Chen F. Li C. et al. (2023). Polysaccharides from *Platycodon grandiflorus* attenuates high-fat diet induced obesity in mice through targeting gut microbiota. *Biomed. Pharmacother.* 166:115318. 10.1016/j.biopha.2023.115318 37572640

[B55] Kiewiet M. B. G. Elderman M. E. El Aidy S. Burgerhof J. G. M. Visser H. Vaughan E. E. et al. (2021). Flexibility of gut microbiota in ageing individuals during dietary fiber long-chain inulin intake. *Mol. Nutr. Food Res.* 65:e2000390. 10.1002/mnfr.202000390 33369019 PMC8138623

[B56] Klimenko N. S. Odintsova V. E. Revel-Muroz A. Tyakht A. V. (2022). The hallmarks of dietary intervention-resilient gut microbiome. *NPJ Biofilms Microbiomes* 8:77. 10.1038/s41522-022-00342-8 36209276 PMC9547895

[B57] Lee P. S. Lu Y. Y. Nagabhushanam K. Ho C. T. Mei H. C. Pan M. H. (2023). Calebin-A prevents HFD-induced obesity in mice by promoting thermogenesis and modulating gut microbiota. *J. Tradit. Complement. Med.* 13 119–127. 10.1016/j.jtcme.2022.01.001 36970457 PMC10037069

[B58] Lei S. He S. Li X. Zheng B. Zhang Y. Zeng H. (2023). Effect of lotus seed resistant starch on small intestinal flora and bile acids in hyperlipidemic rats. *Food Chem* 404(Pt A), 134599. 10.1016/j.foodchem.2022.134599 36444019

[B59] Li C. Wang Y. Huang Z. Zhang Y. Li J. Zhang Q. et al. (2023). Hypoglycemic effect of polysaccharides of *Dendrobium officinale* compound on type 2 diabetic mice. *J. Funct. Foods* 106:105579. 10.1016/j.jff.2023.105579

[B60] Li D. Zhang X. Fan Y. Zhang Y. Tao X. Yang J. (2023). *Lycium barbarum* polysaccharides improved glucose metabolism in prediabetic mice by regulating duodenal contraction. *Nutrients* 15:4437. 10.3390/nu15204437 37892511 PMC10609773

[B61] Li F. Ming J. (2024). Mulberry polyphenols restored both small and large intestinal microflora in db/db mice, potentially alleviating type 2 diabetes. *Food Funct.* 15 8521–8543. 10.1039/d4fo01291g 39058305

[B62] Li L. Li R. Tian Q. Luo Y. Li R. Lin X. et al. (2024). Effects of healthy low-carbohydrate diet and time-restricted eating on weight and gut microbiome in adults with overweight or obesity: feeding RCT. *Cell Rep. Med.* 5:101801. 10.1016/j.xcrm.2024.101801 39454570 PMC11604488

[B63] Li M. Zhao Y. Wang Y. Geng R. Fang J. Kang S. G. et al. (2022). Eugenol, a major component of clove oil, attenuates adiposity, and modulates gut microbiota in high-fat diet-fed mice. *Mol. Nutr. Food Res.* 66:e2200387. 10.1002/mnfr.202200387 36029106

[B64] Li Q. Liu F. Liu J. Liao S. Zou Y. (2019). Mulberry leaf polyphenols and fiber induce synergistic antiobesity and display a modulation effect on gut microbiota and metabolites. *Nutrients* 11:1017. 10.3390/nu11051017 31064150 PMC6567141

[B65] Li Q. Liu W. Zhang H. Chen C. Liu R. Hou H. et al. (2023). α-D-1,3-glucan from Radix *Puerariae thomsonii* improves NAFLD by regulating the intestinal flora and metabolites. *Carbohydr. Polym.* 299:120197. 10.1016/j.carbpol.2022.120197 36876767

[B66] Li R. Xue Z. Li S. Zhou J. Liu J. Zhang M. et al. (2022). Mulberry leaf polysaccharides ameliorate obesity through activation of brown adipose tissue and modulation of the gut microbiota in high-fat diet fed mice. *Food Funct.* 13 561–573. 10.1039/d1fo02324a 34951619

[B67] Li R. Yao Y. Gao P. Bu S. (2020). The therapeutic efficacy of curcumin vs. metformin in modulating the gut microbiota in NAFLD rats: a comparative study. *Front. Microbiol.* 11:555293. 10.3389/fmicb.2020.555293 33584555 PMC7874275

[B68] Li S. You J. Wang Z. Liu Y. Wang B. Du M. et al. (2021). Curcumin alleviates high-fat diet-induced hepatic steatosis and obesity in association with modulation of gut microbiota in mice. *Food Res. Int.* 143:110270. 10.1016/j.foodres.2021.110270 33992371

[B69] Li S. N. Zhang D. L. Wang Z. H. Song W. T. Chen W. B. Hu G. L. et al. (2023). Anti-obesity effects exerted by *Dioscorea opposita* Thunb. polysaccharides in diet-induced obese mice. *Food Sci. Nutr.* 11 6459–6469. 10.1002/fsn3.3588 37823169 PMC10563686

[B70] Li X. Wang Y. Yu C. Yao Y. Chen X. Deng Z. Y. et al. (2022). The signatures of liver metabolomics and gut microbiota in high-fat diet fed mice supplemented with rhododendrol. *Food Funct.* 13 13052–13063. 10.1039/d2fo01214f 36468583

[B71] Li X. Yao Y. Yu C. Wei T. Xi Q. Li J. et al. (2023). Modulation of PPARα-thermogenesis gut microbiota interactions in obese mice administrated with zingerone. *J. Sci. Food Agric.* 103 3065–3076. 10.1002/jsfa.12352 36424723

[B72] Li Y. Huang J. Zhao W. Guan Z. Wang Z. Huang H. et al. (2024). *Polygonatum kingianum* Coll. et Hemsl enzymatic saccharifying extracts alleviate HFD-induced obesity in mice via regulating gut microbiota and AMPK pathways. *Food Biosci.* 60:104094. 10.1016/j.fbio.2024.104094

[B73] Li Y. Li Z. Chen B. Hou Y. Wen Y. Gan L. et al. (2023). Ultrasonic assisted extraction, characterization and gut microbiota-dependent anti-obesity effect of polysaccharide from Pericarpium Citri Reticulatae ‘Chachiensis’. *Ultrason Sonochem.* 95:106383. 10.1016/j.ultsonch.2023.106383 37004413 PMC10457594

[B74] Li Y. Xu W. Zhang F. Zhong S. Sun Y. Huo J. et al. (2020). The gut microbiota-produced indole-3-propionic acid confers the antihyperlipidemic effect of mulberry-derived 1-deoxynojirimycin. *mSystems* 5:e00313-20. 10.1128/msystems.00313-20 33024047 PMC7542557

[B75] Liang J. Li X. Lei W. Tan P. Han M. Li H. et al. (2023). Serum metabolomics combined with 16S rRNA sequencing to reveal the effects of *Lycium barbarum* polysaccharide on host metabolism and gut microbiota. *Food Res. Int.* 165:112563. 10.1016/j.foodres.2023.112563 36869545

[B76] Liu H. Lan Z. Zhang Y. Zhao Z. Wu Y. Tang X. et al. (2025). Metabolomics combined with network pharmacology reveals the effects of ripening stages and edible parts on bioactive ingredients of Luohan Guo (*Siraitia grosvenorii*). *Food Res. Int.* 203:115896. 10.1016/j.foodres.2025.115896 40022403

[B77] Liu H. Xing Y. Wang Y. Ren X. Zhang D. Dai J. et al. (2023). *Dendrobium officinale* polysaccharide prevents diabetes via the regulation of gut microbiota in prediabetic mice. *Foods* 12:2310. 10.3390/foods12122310 37372523 PMC10296996

[B78] Liu L. Han J. Tian H. Liu Z. Wang X. Wang P. et al. (2025). A review on the progress of research on the chemical composition and pharmacological effects of lotus leaf. *Food Chem.* 493(Pt 2):145708. 10.1016/j.foodchem.2025.145708 40753702

[B79] Liu L. Zhao Y. Ming J. Chen J. Zhao G. Chen Z. Y. et al. (2021). Polyphenol extract and essential oil of Amomum tsao-ko equally alleviate hypercholesterolemia and modulate gut microbiota. *Food Funct.* 12 12008–12021. 10.1039/d1fo03082e 34755750

[B80] Liu R. Zhang X. Cai Y. Xu S. Xu Q. Ling C. et al. (2024). Research progress on medicinal components and pharmacological activities of polygonatum sibiricum. *J. Ethnopharmacol.* 328:118024. 10.1016/j.jep.2024.118024 38484952

[B81] Liu W. Yu L. Chen Q. Zhang C. Wang L. Yu N. et al. (2025). Poria cocos polysaccharides alleviate obesity-related adipose tissue insulin resistance via gut microbiota-derived short-chain fatty acids activation of FGF21/PI3K/AKT signaling. *Food Res. Int.* 215:116671. 10.1016/j.foodres.2025.116671 40484558

[B82] Liu Y. X. Song X. M. Dan L. W. Tang J. M. Jiang Y. Deng C. et al. (2024). Astragali Radix: comprehensive review of its botany, phytochemistry, pharmacology and clinical application. *Arch. Pharm. Res.* 47 165–218. 10.1007/s12272-024-01489-y 38493280

[B83] Lu H. Liu P. Zhang X. Bao T. Wang T. Guo L. et al. (2021). Inulin and *Lycium barbarum* polysaccharides ameliorate diabetes by enhancing gut barrier via modulating gut microbiota and activating gut mucosal TLR2+ intraepithelial γδ T cells in rats. *J. Funct. Foods* 79:104407. 10.1016/j.jff.2021.104407

[B84] Lu Q. Li R. Yang Y. Zhang Y. Zhao Q. Li J. (2022). Ingredients with anti-inflammatory effect from medicine food homology plants. *Food Chem.* 368:130610. 10.1016/j.foodchem.2021.130610 34419798

[B85] Lu X. Xie Q. Pan X. Zhang R. Zhang X. Peng G. et al. (2024). Type 2 diabetes mellitus in adults: pathogenesis, prevention and therapy. *Signal Transduct Target Ther.* 9:262. 10.1038/s41392-024-01951-9 39353925 PMC11445387

[B86] Luciani L. Pedrelli M. Parini P. (2024). Modification of lipoprotein metabolism and function driving atherogenesis in diabetes. *Atherosclerosis* 394:117545. 10.1016/j.atherosclerosis.2024.117545 38688749

[B87] Luo J. Chai Y. Zhao M. Guo Q. Bao Y. (2020). Hypoglycemic effects and modulation of gut microbiota of diabetic mice by saponin from *Polygonatum sibiricum*. *Food Funct.* 11 4327–4338. 10.1039/d0fo00428f 32367101

[B88] Luo L. Zhang H. Chen W. Zheng Z. He Z. Wang H. et al. (2023). *Angelica sinensis* polysaccharide ameliorates nonalcoholic fatty liver disease via restoring estrogen-related receptor α expression in liver. *Phytother. Res.* 37 5407–5417. 10.1002/ptr.7982 37563852

[B89] Ma L. La X. Zhang B. Xu W. Tian C. Fu Q. et al. (2023). Total *Astragalus saponins* can reverse type 2 diabetes mellitus-related intestinal dysbiosis and hepatic insulin resistance in vivo. *Front. Endocrinol.* 14:1190827. 10.3389/fendo.2023.1190827 38053727 PMC10694298

[B90] Ma Q. Zhai R. Xie X. Chen T. Zhang Z. Liu H. et al. (2022). Hypoglycemic effects of *Lycium barbarum* polysaccharide in type 2 diabetes mellitus mice via modulating gut microbiota. *Front. Nutr.* 9:916271. 10.3389/fnut.2022.916271 35845787 PMC9280299

[B91] Ma Y. Zhu L. Ke H. Jiang S. Zeng M. (2022). Oyster (*Crassostrea gigas*) polysaccharide ameliorates obesity in association with modulation of lipid metabolism and gut microbiota in high-fat diet fed mice. *Int. J. Biol. Macromol.* 216 916–926. 10.1016/j.ijbiomac.2022.07.100 35868410

[B92] Matsumura Y. Kitabatake M. Kayano S. I. Ito T. (2023). Dietary phenolic compounds: their health benefits and association with the gut microbiota. *Antioxidants* 12:880. 10.3390/antiox12040880 37107256 PMC10135282

[B93] Narrowe A. B. Lemons J. M. S. Mahalak K. K. Firrman J. den Abbeele P. V. Baudot A. et al. (2024). Targeted remodeling of the human gut microbiome using Juemingzi (Senna seed extracts). *Front. Cell Infect. Microbiol.* 14:1296619. 10.3389/fcimb.2024.1296619 38638830 PMC11024242

[B94] Ng C. Y. J. Lai N. P. Y. Ng W. M. Siah K. T. H. Gan R. Y. Zhong L. L. D. (2024). Chemical structures, extraction and analysis technologies, and bioactivities of edible fungal polysaccharides from *Poria cocos*: an updated review. *Int. J. Biol. Macromol.* 261(Pt 1):129555. 10.1016/j.ijbiomac.2024.129555 38278384

[B95] Nie C. Lan J. Guo H. Ouyang Q. Zhao Y. Wang P. et al. (2024). *Codonopsis pilosula* polysaccharides (CPP) intervention alleviates sterigmatocystin (STC)-induced liver injury and gut microbiota dysbiosis. *Int. J. Biol. Macromol.* 275(Pt 2):133190. 10.1016/j.ijbiomac.2024.133190 38897503

[B96] Niu D. An S. Chen X. Bi H. Zhang Q. Wang T. et al. (2020). Corni fructus as a natural resource can treat type 2 diabetes by regulating gut microbiota. *Am. J. Chin. Med.* 48 1385–1407. 10.1142/s0192415x20500688 32907359

[B97] Nychas E. Marfil-Sánchez A. Chen X. Mirhakkak M. Li H. Jia W. et al. (2025). Discovery of robust and highly specific microbiome signatures of non-alcoholic fatty liver disease. *Microbiome* 13:10. 10.1186/s40168-024-01990-y 39810263 PMC11730835

[B98] Pafili K. Roden M. (2021). Nonalcoholic fatty liver disease (NAFLD) from pathogenesis to treatment concepts in humans. *Mol. Metab.* 50:101122. 10.1016/j.molmet.2020.101122 33220492 PMC8324683

[B99] Peng Y. Li Y. Pi Y. Yue X. (2024). Effects of almond (Armeniaca Sibirica L. Lam) polysaccharides on gut microbiota and anti-inflammatory effects on LPS-induced RAW264.7 cells. *Int. J. Biol. Macromol.* 263(Pt 1):130098. 10.1016/j.ijbiomac.2024.130098 38342264

[B100] Peron G. Sut S. Dal Ben S. Voinovich D. Dall’Acqua S. (2020). Untargeted UPLC-MS metabolomics reveals multiple changes of urine composition in healthy adult volunteers after consumption of curcuma longa L. extract. *Food Res. Int.* 127:108730. 10.1016/j.foodres.2019.108730 31882111

[B101] Petrenko V. Sinturel F. Riezman H. Dibner C. (2023). Lipid metabolism around the body clocks. *Prog. Lipid Res.* 91:101235. 10.1016/j.plipres.2023.101235 37187314

[B102] Ping Y. Fan Q. Zhang Y. (2025). Modulating lipid metabolism improves tumor immunotherapy. *J. Immunother. Cancer* 13:e010824. 10.1136/JITC-2024-010824 39904563 PMC11795363

[B103] Portincasa P. Bonfrate L. Vacca M. De Angelis M. Farella I. Lanza E. et al. (2022). Gut microbiota and short chain fatty acids: implications in glucose homeostasis. *Int. J. Mol. Sci.* 23:1105. 10.3390/ijms23031105 35163038 PMC8835596

[B104] Qin B. Yao Y. Zhang J. Wang L. (2023). Bioavailability of coix seed polyphenols in a MKN28/Caco-2 continuous transport model and their lipid-lowering effects via modulating adipocyte differentiation of 3T3-L1 Cells. *J. Agric. Food Chem.* 71 8425–8436. 10.1021/acs.jafc.2c08388 37233613

[B105] Qin T. Chen X. Meng J. Guo Q. Xu S. Hou S. et al. (2024). The role of curcumin in the liver-gut system diseases: from mechanisms to clinical therapeutic perspective. *Crit. Rev. Food Sci. Nutr.* 64 8822–8851. 10.1080/10408398.2023.2204349 37096460

[B106] Reimer R. A. Soto-Vaca A. Nicolucci A. C. Mayengbam S. Park H. Madsen K. L. et al. (2020). Effect of chicory inulin-type fructan-containing snack bars on the human gut microbiota in low dietary fiber consumers in a randomized crossover trial. *Am. J. Clin. Nutr.* 111 1286–1296. 10.1093/ajcn/nqaa074 32320024

[B107] Ren G. Lin Y. Fu Y. Liu F. Wang R. Zhang C. et al. (2025). Multi-omics joint analysis: pachymic acid ameliorated non-alcoholic fatty liver disease by regulating gut microbiota. *Food Res. Int.* 209:116178. 10.1016/j.foodres.2025.116178 40253122

[B108] Ritika R. Shukla S. Sondhi A. Tripathi A. D. Lee J.-K. Patel S. K. et al. (2024). Valorisation of fruit waste for harnessing the bioactive compounds and its therapeutic application. *Trends Food Sci. Technol.* 144:104302. 10.1016/j.tifs.2023.104302

[B109] Roach L. A. Meyer B. J. Fitton J. H. Winberg P. (2022). Improved plasma lipids, anti-inflammatory activity, and microbiome shifts in overweight participants: two clinical studies on oral supplementation with algal sulfated polysaccharide. *Mar. Drugs* 20:500. 10.3390/md20080500 36005503 PMC9410082

[B110] Ross F. C. Patangia D. Grimaud G. Lavelle A. Dempsey E. M. Ross R. P. et al. (2024). The interplay between diet and the gut microbiome: implications for health and disease. *Nat. Rev. Microbiol.* 22 671–686. 10.1038/s41579-024-01068-4 39009882

[B111] Saad M. J. A. Santos A. (2025). The microbiota and evolution of obesity. *Endocr. Rev.* 46 300–316. 10.1210/endrev/bnae033 39673174 PMC11894537

[B112] Sang T. Guo C. Guo D. Wu J. Wang Y. Wang Y. et al. (2021). Suppression of obesity and inflammation by polysaccharide from sporoderm-broken spore of *Ganoderma lucidum* via gut microbiota regulation. *Carbohydr. Polym.* 256:117594. 10.1016/j.carbpol.2020.117594 33483079

[B113] Sanz Y. Cryan J. F. Deschasaux-Tanguy M. Elinav E. Lambrecht R. Veiga P. (2025). The gut microbiome connects nutrition and human health. *Nat. Rev. Gastroenterol. Hepatol.* 22 534–555. 10.1038/s41575-025-01077-5 40468006

[B114] Schoultz I. Claesson M. J. Dominguez-Bello M. G. Fåk Hållenius F. Konturek P. Korpela K. et al. (2025). Gut microbiota development across the lifespan: disease links and health-promoting interventions. *J. Intern. Med.* 297 560–583. 10.1111/joim.20089 40270478 PMC12087861

[B115] Shan H. Fan S. Li Q. Liang R. Chen Z. Wang S. et al. (2025). Systematic interrogation of functional genes underlying cholesterol and lipid homeostasis. *Genome Biol.* 26:59. 10.1186/s13059-025-03531-8 40098013 PMC11912599

[B116] Shao W. Xiao C. Yong T. Zhang Y. Hu H. Xie T. et al. (2022). A polysaccharide isolated from *Ganoderma lucidum* ameliorates hyperglycemia through modulating gut microbiota in type 2 diabetic mice. *Int. J. Biol. Macromol.* 197 23–38. 10.1016/j.ijbiomac.2021.12.034 34920067

[B117] Shi Y. Si D. Chen D. Zhang X. Han Z. Yu Q. et al. (2023). Bioactive compounds from *Polygonatum genus* as anti-diabetic agents with future perspectives. *Food Chem.* 408:135183. 10.1016/j.foodchem.2022.135183 36566543

[B118] Song J. Liu Q. Hao M. Zhai X. Chen J. (2023). Effects of neutral polysaccharide from *Platycodon grandiflorum* on high-fat diet-induced obesity via the regulation of gut microbiota and metabolites. *Front. Endocrinol.* 14:1078593. 10.3389/fendo.2023.1078593 36777345 PMC9908743

[B119] Song Q. Cheng S. W. Li D. Cheng H. Lai Y. S. Han Q. et al. (2022). Gut microbiota mediated hypoglycemic effect of *Astragalus membranaceus* polysaccharides in db/db mice. *Front. Pharmacol.* 13:1043527. 10.3389/fphar.2022.1043527 36452223 PMC9703139

[B120] Song Q. Zou J. Li D. Cheng S. W. Li K. L. S. Yang X. et al. (2024). Gastrointestinal metabolism of *Astragalus membranaceus* polysaccharides and its related hypoglycemic mechanism based on gut microbial transformation. *Int. J. Biol. Macromol.* 280(Pt 3):135847. 10.1016/j.ijbiomac.2024.135847 39307509

[B121] Su H. Xie L. Xu Y. Ke H. Bao T. Li Y. et al. (2020). Pelargonidin-3-O-glucoside derived from wild raspberry exerts antihyperglycemic effect by inducing autophagy and modulating gut microbiota. *J. Agric. Food Chem.* 68 13025–13037. 10.1021/acs.jafc.9b03338 31322351

[B122] Tan Y. Y. Yue S. R. Lu A. P. Zhang L. Ji G. Liu B. C. et al. (2022). The improvement of nonalcoholic steatohepatitis by *Poria cocos* polysaccharides associated with gut microbiota and NF-κB/CCL3/CCR1 axis. *Phytomedicine* 103:154208. 10.1016/j.phymed.2022.154208 35691078

[B123] Tian F. Chen T. Xu W. Fan Y. Feng X. Huang Q. et al. (2023). Curcumin compensates GLP-1 deficiency via the microbiota-bile acids axis and modulation in functional crosstalk between TGR5 and FXR in ob/ob Mice. *Mol. Nutr. Food Res.* 67:e2300195. 10.1002/mnfr.202300195 37712101

[B124] Tian H. An L. Wang P. Zhang X. Gao W. Li X. (2025). Review of *Astragalus membranaceus* polysaccharides: extraction process, structural features, bioactivities and applications. *Chin. Herb. Med.* 17 56–69. 10.1016/j.chmed.2024.09.004 39949812 PMC11814244

[B125] Tilg H. Adolph T. E. Dudek M. Knolle P. (2021). Non-alcoholic fatty liver disease: the interplay between metabolism, microbes and immunity. *Nat. Metab.* 3 1596–1607. 10.1038/s42255-021-00501-9 34931080

[B126] Tosi N. Bragazzi N. L. Mignogna C. Treccani M. Bresciani L. Vauzour D. et al. (2025). The impact of single nucleotide polymorphisms on the absorption, distribution, metabolism, and excretion of dietary (poly)phenols: a critical systematic review. *Food Funct.* 16 9259–9281. 10.1039/D5FO03349G 41258761 PMC12628764

[B127] Unamuno X. Gómez-Ambrosi J. Rodríguez A. Becerril S. Frühbeck G. Catalán V. (2018). Adipokine dysregulation and adipose tissue inflammation in human obesity. *Eur. J. Clin. Invest.* 48:e12997. 10.1111/eci.12997 29995306

[B128] Wan M. Li Q. Lei Q. Zhou D. Wang S. (2022). Polyphenols and polysaccharides from *Morus alba* L. Fruit attenuate high-fat diet-induced metabolic syndrome modifying the gut microbiota and metabolite profile. *Foods* 11:1818. 10.3390/foods11121818 35742014 PMC9223293

[B129] Wan Y. Xia J. Xu J. F. Chen L. Yang Y. Wu J. J. et al. (2022). Nuciferine, an active ingredient derived from lotus leaf, lights up the way for the potential treatment of obesity and obesity-related diseases. *Pharmacol. Res.* 175:106002. 10.1016/j.phrs.2021.106002 34826599

[B130] Wang L. Chen W. Tian Y. Duan X. Yuan Y. Wang N. et al. (2022a). Preventive effects of sesamol on deep-frying oil-induced liver metabolism disorders by altering gut microbiota and protecting gut barrier integrity. *Mol. Nutr. Food Res.* 66:e2101122. 10.1002/mnfr.202101122 35184393

[B131] Wang L. Qiu Y. Gu H. Gan M. Zhu Y. Zhu K. et al. (2022b). Regulation of adipose thermogenesis and its critical role in glucose and lipid metabolism. *Int. J. Biol. Sci.* 18 4950–4962. 10.7150/ijbs.75488 35982903 PMC9379406

[B132] Wang S. Cui K. Liu J. Hu J. Yan K. Xiao P. et al. (2022c). Mogroside-rich extract from siraitia grosvenorii fruits ameliorates high-fat diet-induced obesity associated with the modulation of gut microbiota in mice. *Front. Nutr.* 9:870394. 10.3389/fnut.2022.870394 35769373 PMC9234556

[B133] Wang S. Zuo Z. Ye B. Zhang L. Cheng Y. Xie S. et al. (2023). Microbiome-metabolomic analysis reveals beneficial effects of dietary kelp resistant starch on intestinal functions of hybrid snakeheads (*Channa maculata* ? × *Channa argus* ?). *Antioxidants* 12:1631. 10.3390/antiox12081631 37627626 PMC10451247

[B134] Wang X. Zhang D. Jiang H. Zhang S. Pang X. Gao S. et al. (2020). Gut microbiota variation with short-term intake of ginger juice on human health. *Front. Microbiol.* 11:576061. 10.3389/fmicb.2020.576061 33708178 PMC7940200

[B135] Wang Y. Yao W. Li B. Qian S. Wei B. Gong S. et al. (2020). Nuciferine modulates the gut microbiota and prevents obesity in high-fat diet-fed rats. *Exp. Mol. Med.* 52 1959–1975. 10.1038/s12276-020-00534-2 33262480 PMC8080667

[B136] Wang Z. Yang T. Zeng M. Wang Z. Chen Q. Chen J. et al. (2023). Miquelianin in Folium Nelumbinis extract promotes white-to-beige fat conversion via blocking AMPK/DRP1/mitophagy and modulating gut microbiota in HFD-fed mice. *Food Chem. Toxicol.* 181:114089. 10.1016/j.fct.2023.114089 37804915

[B137] Watson A. W. Houghton D. Avery P. J. Stewart C. Vaughan E. E. Meyer P. D. et al. (2019). Changes in stool frequency following chicory inulin consumption, and effects on stool consistency, quality of life and composition of gut microbiota. *Food Hydrocoll.* 96 688–698. 10.1016/j.foodhyd.2019.06.006 31680713 PMC6686634

[B138] Wedan R. J. Longenecker J. Z. Nowinski S. M. (2024). Mitochondrial fatty acid synthesis is an emergent central regulator of mammalian oxidative metabolism. *Cell Metab.* 36 36–47. 10.1016/j.cmet.2023.11.017 38128528 PMC10843818

[B139] Westman E. C. (2021). Type 2 diabetes mellitus: a pathophysiologic perspective. *Front. Nutr.* 8:707371. 10.3389/fnut.2021.707371 34447776 PMC8384107

[B140] Wu M. Lyu Y. Xu H. Luo H. Yin X. Zheng H. (2024). Raspberry polysaccharides attenuate hepatic inflammation and oxidative stress in diet-induced obese mice by enhancing butyrate-mediated intestinal barrier function. *Int. J. Biol. Macromol.* 262(Pt 2):130007. 10.1016/j.ijbiomac.2024.130007 38340928

[B141] Wu Q. H. Luo L. Luo Q. Hong T. Xu L. Ma Q. et al. (2023). Dietary ginger polysaccharides (Gps) improve symptoms in hyperlipidemia rats via alterations in gut microbiota. *Heliyon* 9:e17534. 10.1016/j.heliyon.2023.e17534 37456047 PMC10345252

[B142] Wu S. Chen X. Cai R. Chen X. Zhang J. Xie J. et al. (2023). Sulfated Chinese yam polysaccharides alleviate LPS-induced acute inflammation in mice through modulating intestinal microbiota. *Foods* 12:1772. 10.3390/foods12091772 37174310 PMC10178587

[B143] Wu Y. L. Lin Z. J. Li C. C. Lin X. Shan S. K. Guo B. et al. (2023). Epigenetic regulation in metabolic diseases: mechanisms and advances in clinical study. *Signal Transduct. Target Ther.* 8:98. 10.1038/s41392-023-01333-7 36864020 PMC9981733

[B144] Xia L. Li C. Zhao J. Sun Q. Mao X. (2025). Rebalancing immune homeostasis in combating disease: the impact of medicine food homology plants and gut microbiome. *Phytomedicine* 136:156150. 10.1016/j.phymed.2024.156150 39740376

[B145] Xia T. Liu C. S. Hu Y. N. Luo Z. Y. Chen F. L. Yuan L. X. et al. (2021). Coix seed polysaccharides alleviate type 2 diabetes mellitus via gut microbiota-derived short-chain fatty acids activation of IGF1/PI3K/AKT signaling. *Food Res. Int.* 150(Pt A):110717. 10.1016/j.foodres.2021.110717 34865748

[B146] Xian Y. Fan R. Shao J. Mulcahy Toney A. Chung S. Ramer-Tait A. E. (2021). Polyphenolic fractions isolated from red raspberry whole fruit, pulp, and seed differentially alter the gut microbiota of mice with diet-induced obesity. *J. Funct. Foods* 76:104288. 10.1016/j.jff.2020.104288

[B147] Xiong R. G. Zhou D. D. Wu S. X. Huang S. Y. Saimaiti A. Yang Z. J. et al. (2022). Health benefits and side effects of short-chain fatty acids. *Foods* 11:2863. 10.3390/foods11182863 36140990 PMC9498509

[B148] Xu S. Lu F. Gao J. Yuan Y. (2024). Inflammation-mediated metabolic regulation in adipose tissue. *Obes. Rev.* 25:e13724. 10.1111/obr.13724 38408757

[B149] Xuan W. Ou Y. Chen W. Huang L. Wen C. Huang G. et al. (2023). *Faecalibacterium prausnitzii* improves lipid metabolism disorder and insulin resistance in type 2 diabetic mice. *Br. J. Biomed. Sci.* 80:10794. 10.3389/bjbs.2023.10794 37025162 PMC10070466

[B150] Xue H. Tang Y. Zha M. Xie K. Tan J. (2025). The structure-function relationships and interaction between polysaccharides and intestinal microbiota: a review. *Int. J. Biol. Macromol.* 291:139063. 10.1016/j.ijbiomac.2024.139063 39710020

[B151] Yang M. Lv J. Yang J. Yang S. Wang F. Wang Y. et al. (2023). Effects of *Codonopsis pilosula* crude polysaccharides by hypoglycemic and modulating gut microbiome in a high-fat diet and streptozotocin-induced mouse model of T2DM. *J. Funct. Foods* 111:105893. 10.1016/j.jff.2023.105893

[B152] Yang M. Yin Y. Wang F. Zhang H. Ma X. Yin Y. et al. (2021). Supplementation with *Lycium barbarum* polysaccharides reduce obesity in high-fat diet-fed mice by modulation of gut microbiota. *Front. Microbiol.* 12:719967. 10.3389/fmicb.2021.719967 34512598 PMC8427603

[B153] Yang T. Zhou W. Xu W. Ran L. Yan Y. Lu L. et al. (2022). Modulation of gut microbiota and hypoglycemic/hypolipidemic activity of flavonoids from the fruits of *Lycium barbarum* on high-fat diet/streptozotocin-induced type 2 diabetic mice. *Food Funct.* 13 11169–11184. 10.1039/d2fo01268e 36218053

[B154] Yang Y. Chang Y. Wu Y. Liu H. Liu Q. Kang Z. et al. (2021). A homogeneous polysaccharide from *Lycium barbarum*: structural characterizations, anti-obesity effects and impacts on gut microbiota. *Int. J. Biol. Macromol.* 183 2074–2087. 10.1016/j.ijbiomac.2021.05.209 34097961

[B155] Yasmin A. Muhammad Ijaz A. Tuerxunayi A. Junhao W. Minjie Z. Fengqin F. et al. (2023). 6-Gingerol, an active ingredient of ginger, reshapes gut microbiota and serum metabolites in HFD-induced obese mice. *J. Funct. Foods* 109:105783. 10.1016/j.jff.2023.105783

[B156] Yuan H. Xu G. Liu J. Yan Y. Zhao S. Cai F. et al. (2025). *Astragalus mongholicus* polysaccharides alleviate insulin resistance through modulation of PI3K/AKT, TLR4/NF-kB signaling pathway and microbiota in rats with Type 2 Diabetes Mellitus. *J. Tradit. Complement. Med.* 15 274–285. 10.1016/j.jtcme.2024.05.007 40486274 PMC12143329

[B157] Yuan T. Yin Z. Yan Z. Hao Q. Zeng J. Li L. et al. (2020). *Tetrahydrocurcumin ameliorates* diabetes profiles of db/db mice by altering the composition of gut microbiota and up-regulating the expression of GLP-1 in the pancreas. *Fitoterapia* 146:104665. 10.1016/j.fitote.2020.104665 32531320

[B158] Zeng H. He S. Xiong Z. Su J. Wang Y. Zheng B. et al. (2023). Gut microbiota-metabolic axis insight into the hyperlipidemic effect of lotus seed resistant starch in hyperlipidemic mice. *Carbohydr. Polym.* 314:120939. 10.1016/j.carbpol.2023.120939 37173019

[B159] Zhang C. Pi X. Li X. Huo J. Wang W. (2024). Edible herbal source-derived polysaccharides as potential prebiotics: composition, structure, gut microbiota regulation, and its related health effects. *Food Chem.* 458:140267. 10.1016/j.foodchem.2024.140267 38968717

[B160] Zhang Q. Bai Y. Wang W. Li J. Zhang L. Tang Y. et al. (2023). Role of herbal medicine and gut microbiota in the prevention and treatment of obesity. *J. Ethnopharmacol.* 305:116127. 10.1016/j.jep.2022.116127 36603782

[B161] Zhang X. Jia L. Ma Q. Zhang X. Chen M. Liu F. et al. (2024). Astragalus polysaccharide modulates the gut microbiota and metabolites of patients with type 2 diabetes in an in vitro fermentation model. *Nutrients* 16:1698. 10.3390/nu16111698 38892631 PMC11174380

[B162] Zhang X. Zhao A. Sandhu A. K. Edirisinghe I. Burton-Freeman B. M. (2022). Red raspberry and fructo-oligosaccharide supplementation, metabolic biomarkers, and the gut microbiota in adults with prediabetes: a randomized crossover clinical trial. *J. Nutr.* 152 1438–1449. 10.1093/jn/nxac037 35421233

[B163] Zhang Y. Miao R. Ma K. Zhang Y. Fang X. Wei J. et al. (2023). Effects and mechanistic role of mulberry leaves in treating diabetes and its complications. *Am. J. Chin. Med.* 51 1711–1749. 10.1142/S0192415X23500775 37646143

[B164] Zhang Y. Peng Y. Zhao L. Zhou G. Li X. (2021a). Regulating the gut microbiota and SCFAs in the faeces of T2DM rats should be one of antidiabetic mechanisms of mogrosides in the fruits of *Siraitia grosvenorii*. *J. Ethnopharmacol.* 274:114033. 10.1016/j.jep.2021.114033 33741440

[B165] Zhang Y. Zhao N. Yang L. Hong Z. Cai B. Le Q. et al. (2021b). Insoluble dietary fiber derived from brown seaweed *Laminaria japonica* ameliorate obesity-related features via modulating gut microbiota dysbiosis in high-fat diet-fed mice. *Food Funct.* 12 587–601. 10.1039/d0fo02380a 33350422

[B166] Zhao X. Fu Z. Yao M. Cao Y. Zhu T. Mao R. et al. (2022). Mulberry (*Morus alba* L.) leaf polysaccharide ameliorates insulin resistance- and adipose deposition-associated gut microbiota and lipid metabolites in high-fat diet-induced obese mice. *Food Sci. Nutr.* 10 617–630. 10.1002/fsn3.2689 35154697 PMC8825736

[B167] Zheng H. Ji H. Fan K. Xu H. Huang Y. Zheng Y. et al. (2022). Targeting gut microbiota and host metabolism with dendrobium officinale dietary fiber to prevent obesity and improve glucose homeostasis in diet-induced obese mice. *Mol. Nutr. Food Res.* 66:e2100772. 10.1002/mnfr.202100772 35225418

[B168] Zheng L. Lu Z. Ma Y. Cui P. Zhang X. Gan J. et al. (2025). Hawthorn total flavonoids ameliorate hyperlipidemia through AMPK/SREBP1-c and PPARα/PGC-1α/CPT-1A pathway activation and gut microbiota modulation. *J. Sci. Food Agric.* 105 4326–4337. 10.1002/jsfa.14188 40013442

[B169] Zheng W. Duan M. Jia J. Song S. Ai C. (2021). Low-molecular alginate improved diet-induced obesity and metabolic syndrome through modulating the gut microbiota in BALB/c mice. *Int. J. Biol. Macromol.* 187 811–820. 10.1016/j.ijbiomac.2021.08.003 34363822

[B170] Zhi N. Chang X. Zha L. Zhang K. Wang J. Gui S. (2025). Platycodonis radix polysaccharides suppress progression of high-fat-induced obesity through modulation of intestinal microbiota and metabolites. *Phytomedicine* 142:156653. 10.1016/j.phymed.2025.156653 40354675

[B171] Zhong M. Yan Y. Yuan H. Rong A. Xu G. Cai F. et al. (2022). Astragalus mongholicus polysaccharides ameliorate hepatic lipid accumulation and inflammation as well as modulate gut microbiota in NAFLD rats. *Food Funct.* 13 7287–7301. 10.1039/d2fo01009g 35726797

[B172] Zhong Y. Song B. Zheng C. Zhang S. Yan Z. Tang Z. et al. (2020). Flavonoids from mulberry leaves alleviate lipid dysmetabolism in high fat diet-fed mice: involvement of gut microbiota. *Microorganisms* 8:860. 10.3390/microorganisms8060860 32517288 PMC7355566

[B173] Zhou D. Chen Y. Liu P. Zhu K. Holder-Haynes J. Lloyd S. J. et al. (2025). Genetics-nutrition interactions control diurnal enhancer-promoter dynamics and liver lipid metabolism. *Cell Metab.* 37 1961–79.e7. 10.1016/j.cmet.2025.07.010 40858101 PMC12923262

[B174] Zhou R. He D. Zhang H. Xie J. Zhang S. Tian X. et al. (2023). Ginsenoside Rb1 protects against diabetes-associated metabolic disorders in Kkay mice by reshaping gut microbiota and fecal metabolic profiles. *J. Ethnopharmacol.* 303:115997. 10.1016/j.jep.2022.115997 36509256

[B175] Zhou W. Liu P. Xu W. Ran L. Yan Y. Lu L. et al. (2023). A purified fraction of polysaccharides from the fruits of *Lycium barbarum* L. improves glucose homeostasis and intestinal barrier function in high-fat diet-fed mice. *Food Funct.* 14 5311–5325. 10.1039/d3fo00262d 37203380

[B176] Zhou W. Yang T. Xu W. Huang Y. Ran L. Yan Y. et al. (2022). The polysaccharides from the fruits of *Lycium barbarum* L. confer anti-diabetic effect by regulating gut microbiota and intestinal barrier. *Carbohydr. Polym.* 291:119626. 10.1016/j.carbpol.2022.119626 35698418

[B177] Zhou X. Peng Z. Liao Y. Li D. Xu S. Wen Y. et al. (2023). Weight reduction effect of alginate associated with gut microbiota and bile acids: a double-blind and randomized trial. *J. Funct. Foods* 108:105774. 10.1016/j.jff.2023.105774

[B178] Zmora N. Suez J. Elinav E. (2019). You are what you eat: diet, health and the gut microbiota. *Nat. Rev. Gastroenterol. Hepatol.* 16 35–56. 10.1038/s41575-018-0061-2 30262901

